# Loss of progesterone receptor is associated with distinct tyrosine kinase profiles in breast cancer

**DOI:** 10.1007/s10549-020-05763-7

**Published:** 2020-07-24

**Authors:** Andliena Tahiri, Xavier Tekpli, Somisetty V. Satheesh, Rik DeWijn, Torben Lüders, Ida R. Bukholm, Antoni Hurtado, Jürgen Geisler, Vessela N. Kristensen

**Affiliations:** 1 Department of Clinical Molecular Biology (EpiGen), Akershus University Hospital, Lørenskog, Norway; 2 Department of Medical Genetics, Oslo University Hospital, Oslo, Norway; 3 Institute of Clinical Medicine, University of Oslo, Oslo, Norway; 4 Hamilton Nordics, Slattum, Norway; 5 PamGene International B.V., ‘s-Hertogenbosch, The Netherlands; 6 Helgelandssykehuset HF and Norwegian University of Life Sciences (NMBU), Ås, Norway; 7 Department of Biomedical Sciences, Faculty of Medicine, University of Barcelona, Casanova, Barcelona, Spain; 8 August Pi I Sunyer Research Center (IDIBAPS), Barcelona, Spain; 9 Department of Oncology, Akershus University Hospital, Lørenskog, Norway

**Keywords:** Breast cancer, ER, HER2, PI3K, PR, Tyrosine kinase

## Abstract

**Purpose:**

The aim of this study was to assess protein tyrosine kinase profiles in primary breast cancer samples in correlation with the distinct hormone and growth receptor profiles ER, PR, and HER2.

**Experimental design:**

Pamchip® microarrays were used to measure the phosphorylation of 144 tyrosine kinase substrates in 29 ER+ breast cancer samples and cell lines MCF7, BT474 and ZR75-1. mRNA expression data from the METABRIC cohort and publicly available PR chip-sequencing data were used for validation purposes, together with RT-PCR.

**Results:**

In ER+ breast tumors and cell lines, we observed that the loss of PR expression correlated to higher kinase activity in samples and cell lines that were HER2−. A number of kinases, representing mostly proteins within the PI3K/AKT pathway, were identified as responsible for the differential phosphorylation between PR− and PR+ in ER+/HER2− tumors. We used the METABRIC cohort to analyze mRNA expression from 977 ER+/HER2− breast cancers. Twenty four kinase-encoding genes were identified as differentially expressed between PR+ and PR−, dividing ER+/HER2− samples in two distinct clusters with significant differences in survival (*p* < 0.05). Four kinase genes, LCK, FRK, FGFR4, and MST1R, were identified as potential direct targets of PR.

**Conclusions:**

Our results suggest that the PR status has a profound effect on tyrosine kinases, especially for FGFR4 and LCK genes, in ER+/HER2− breast cancer patients. The influence of these genes on the PI3K/AKT signaling pathway may potentially lead to novel drug targets for ER+/PR− breast cancer patients.

**Electronic supplementary material:**

The online version of this article (10.1007/s10549-020-05763-7) contains supplementary material, which is available to authorized users.

## Introduction

Breast cancer is a complex disease, and depending on the molecular profiles of the tumor, it can be classified into several distinct intrinsic subtypes with different prognostic outcome [1, 2]. The overall tumor biology of breast cancer subtypes is highly dependent on the expression of estrogen receptor (ER), progesterone receptor (PR) and the human epidermal growth factor receptor 2 (HER2). One of the most fundamental clinical distinctions between breast cancer subtypes is whether the tumor responds to growth signaling through the hormonal (ER/PR) or HER2 receptors, as these tumors can be targeted with modern anti-hormonal or anti-HER2 therapy, respectively. Positivity for ER, PR and HER2 has therefore become highly important in the clinical management of breast cancer, both in the neoadjuvant/adjuvant and metastatic setting of the disease. Approximately 70% breast cancers are hormone receptor (HR) positive, meaning that they either express ER and/or PR to some extent [3]. In ER+ breast cancers, PR is often used as a positive prognostic marker of disease outcome [4], but the role of PR signaling in these cancers, still remains unclear. In advanced breast cancer, around 65% of all HR-positive breast cancer patients respond well to endocrine therapy [5, 6], whereas patients that are ER positive and PR negative (ER+/PR−) have significantly worse prognosis [7–9]. The PR is an ER-regulated gene, and ER+ tumors are usually also PR+, whereas ER- tumors are usually PR−. Therefore, single HR-positive (i.e., ER+/PR− or ER−/PR+) tumors represent only a small subgroup of breast cancer cases [10]. While the majority of tumors are HR positive, nearly 15–20% of all breast cancer patients exhibit HER2 overexpression or HER2/*neu* gene amplification. HER2− positive (HER2+) tumors are very heterogeneous, and in many cases, the overexpression of HER2 is associated with the loss of both ER and PR expression, but for 10% of breast cancers, both ER and HER2 are co-expressed [11]. Women with both HER2- and HR-positive cancers do have a tendency to exhibit early resistance towards endocrine therapy [12]. However, the prognostic impact of HER2 in breast cancer and the effectiveness of its drug target are well established in breast cancer patients, whereas the role of other protein tyrosine kinases (PTKs) in the loop of networks in ER+ breast cancer patients is rather unknown. PTKs have for a long time been considered as potent targets for the treatment of cancer as they are important mediators of signaling cascades, and may facilitate tumor progression. Nearly half of the tyrosine kinase complement is deregulated in cancer [13], which has led to the recognition of deregulated PTKs as potential biomarkers for stratifying patients to personalized treatments [14, 15]. In this study, we report a comprehensive analysis of tyrosine kinase activity in HR-positive breast tumors that are either HER2+ or HER2−. To our knowledge, profiling of a large number of PTKs in primary breast cancer specimens has not previously been performed, and will add valuable information regarding the aspect of molecular profiles of breast tumors, and will be important in identifying important biomarkers for clinical intervention.

## Materials and methods

### Tissue specimens

All breast cancer tissue specimens were collected at Akershus University Hospital between 2012 and 2014. After surgical removal, tumor tissues were flash frozen in liquid nitrogen prior to transportation and then stored at − 80 °C. A specialized breast cancer pathologist performed histopathological examination of the tumor tissue. In total, we included 32 breast cancer samples for a comprehensive PTK profiling. Subjects involved in the study are mostly HR positive (ER+ and/or PR+) and either HER2+ or HER2−, based on pathological assessment (See supplementary file 1 for detailed sample information). Three samples were excluded from subsequent analysis due to ER negativity. Each specimen was sectioned with a cryotome to get ~ 80 mg of tissue that was used for PTK phosphorylation profiling.

### Cell lines

Three ER+ breast cancer cell lines were used for PTK profiling: MCF7, ZR75-1 and BT474. The cell lines have previously been described [16, 17], and western blotting was performed to assess ER, PR and HER2 expression (see supplementary file 2). The breast cancer cell lines were obtained from American Type Culture Collection (ATCC, Manassas, VA). ZR75-1 cells were grown in RPMI 1640 (21875091; Invitrogen). MCF7 and BT474 cells were plated in DMEM (4.5 g/l glucose; DMEM; 41966-052; Invitrogen), supplemented with 10% FBS, 4 mM l-glutamine, 0.01 mg/ml insulin, 1 mM Na-pyruvate. The cells were grown and treated in the presence and absence of 10 nM of 17β-estradiol for 10 min, as previously described [18].

### Cell lysis and protein level determination

All breast tumor samples and cell line pellets were lysed with the mammalian protein extraction reagent (M-PER) buffer (Pierce Biotechnology, Inc., Rockford, IL), supplemented with 1:100 Halt Phosphatase and Protease inhibitor cocktail (EDTA free) (Pierce Biotechnology, Inc). The protein concentration of lysates was determined using the BCA assay (Pierce Biotechnology, Inc.).

### Pamchip protein tyrosine kinase arrays and statistical analysis

PTK profiling was performed using the Tyrosine Kinase PamChip® Array for Pamstation®12 (PamGene International B.V., ‘s-Hertogenbosch, The Netherlands) as previously described [19]. For each experiment, 10 µg of protein lysate was added to the reaction mixture. Each run was performed with 12 different tumor samples, and the run was repeated three times to assess technical variation across samples. For the subsequent data analysis, the end-level kinetic log2 value for each peptide was used. Saturated spots were removed from the analysis, as well as peptides that had low signals in the majority of the samples. Here, we used a cut-off of 25%, meaning that only peptides that were detected in at least 25% of the samples were included, leaving us with a total of 104 peptides for subsequent analysis. Technical replicates were averaged and log2 transformed, and replicates with high variation among replicates were excluded from the analysis. For the experiments involving cell lines, we compared PTK profiling of lysates of untreated cells (E0) against cells treated with 17β estradiol for 10 min (E10). PTK profiling with cell lines was performed using two technical replicates per condition, and the run was performed in a single experiment.

### Upstream kinase analysis

BioNavigator software v.6 (PamGene) was used to perform upstream PTK analysis by comparing differences between ER+/HER2−/PR+ and ER+/HER2−/PR− samples, and linking them to the putative upstream kinases responsible for the difference in phosphorylation between the two groups. The method uses in silico predictions to identify the upstream kinases through Kinexus Kinase Predictor (www.phosphonet.ca), as previously described [20, 21]. The program calculates a significance score or specificity score of a kinase Q = − 10log [max(*m*/*M*, 1/*M*)], where *m* is the number of times out of *M* permutations that | *τ*p | >| *τ* |, where *τ*p is the value of the difference statistic obtained after permutation of the sample or peptide labels, respectively. Kinases are then ranked based on the sum of both scores [21].

### RT-PCR

Total RNA was extracted from all specimens using TRIzol (Invitrogen, Carlsbad, CA) in combination with RNeasy Mini Kit (Qiagen, Valencia, CA). The method combines phenol/guanidine-based lysis and silica membrane column purification of total RNA. RT-PCR was performed in triplicates for 24 breast cancer tissue samples, and for cell lines, using the previously described validation strategies [22]. Primers used for this project were, fyn-related Src family tyrosine kinase (FRK), fibroblast growth factor receptor 4 (FGFR4), Lymphocyte Cell-Specific Protein Tyrosine Kinase (LCK), Macrophage Stimulating 1 Receptor (MST1R), ERBB2, Estrogen Receptor 1 (ESR1), and the control genes ubiquitin C (UBC) and TATA box Binding Protein (TBP) (see supplementary file 3 for primer sequence).

### METABRIC cohort

The breast cancer mRNA expression dataset disclosed by the Molecular Taxonomy of Breast Cancer International Consortium (METABRIC) study (EGAS00000000083) was used [23]. Only ER+ breast cancer samples (n = 1052), divided into four different categories, were used for analysis. We have analyzed ER+/HER2−/PR+ (n = 680) against ER+/HER2−/PR- (n = 297) samples, and ER+/HER2+/PR+ samples (n = 33) against ER+/HER2+/PR− (n = 42) samples. There were 977 ER+/HER2− samples. Furthermore, 24,368 genes were identified to be expressed above background levels and used for further analysis.

### Chip-sequencing data

ChIP-sequencing data from MCF7 cell line were obtained from a previously published dataset [24] where MCF7 cells were treated with either 100 nM progesterone (PG2) or ethanol (vehicle) for three constitutive days.

### Data analysis

Signal quantification on phosphorylated peptides and quality control was performed in BioNavigator software v.6 (PamGene International, ‘s-Hertogenbosch, the Netherlands) as previously described [21], and the data were exported to R. All statistical analyses were consequently performed in the R software (r-project.org), unless otherwise specified. The pheatmap package was used for heatmaps and clustering, and differential expression (*p* < 0.05) was calculated in microarray data using R software. Pathway analysis was performed using DAVID Bioinformatics resources [25, 26] and Gene Set Enrichment Analysis (GSEA) [27, 28]. The microarray data are deposited in GEO database (GEO accession: pending).

## Results

### Protein tyrosine kinase activity profiling in ER-positive breast cancer samples and cell lines

**Fig. 1:**
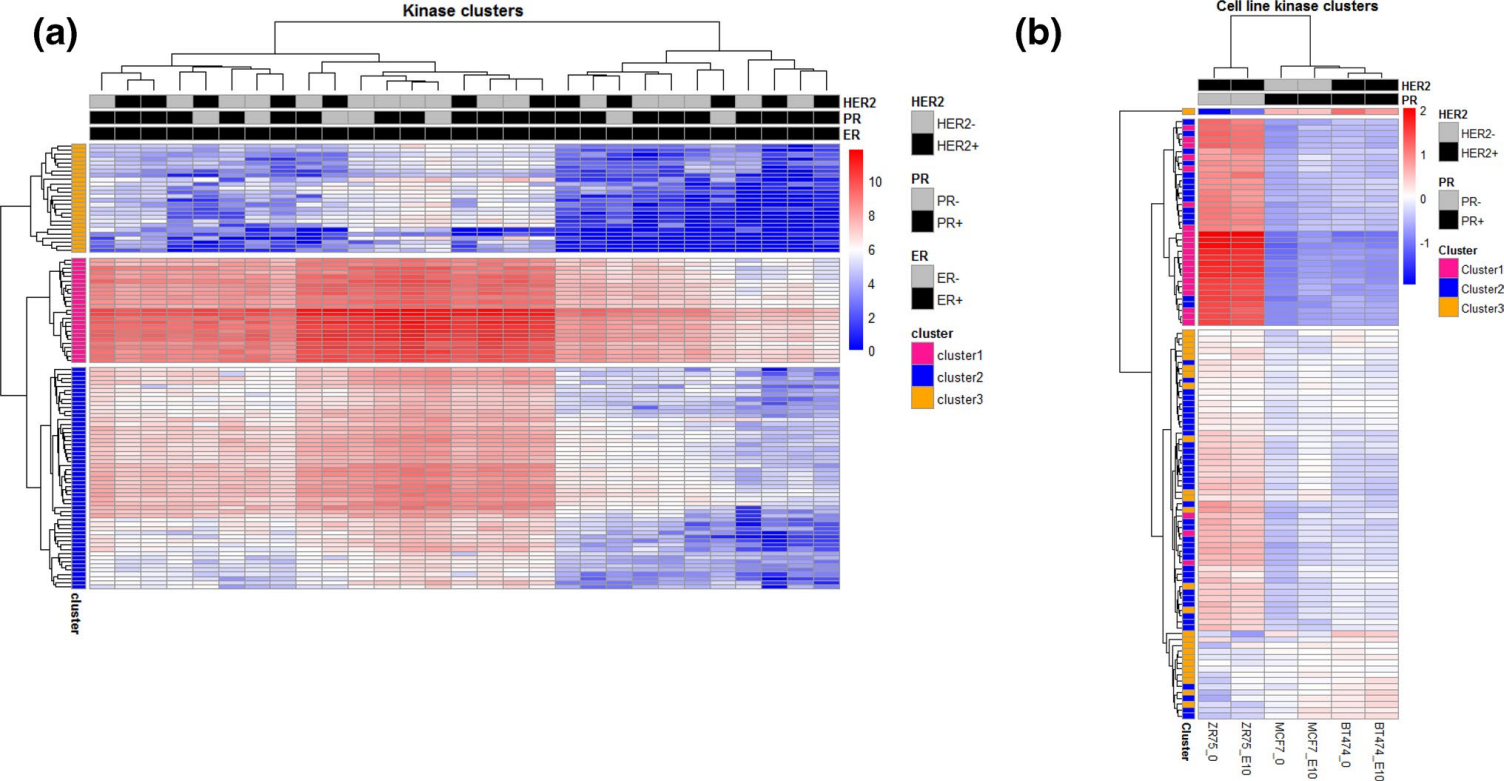
Unsupervised analysis showing phosphorylation profiles of tyrosine kinase substrates in **a** 29 primary ER-positive breast cancer tumors and **b** ER-positive breast cancer cell lines, MCF7, BT474, and ZR75-1, before (E0) and after (E10) treatment with estradiol (E). The heatmap visualizes log2-transformed signal intensities retrieved from tyrosine kinase substrate arrays incubated with sample lysates, in which the samples (horizontal axis) and 104 peptides (vertical axis) are sorted according to principles of hierarchical clustering. Red corresponds to higher and blue to lower kinase substrate phosphorylation levels. *ER−* estrogen receptor, *PR−* progesterone receptor, and *HER2−* human epidermal growth factor receptor 2

Microarrays were used to profile the phosphorylation of substrate kinases in the primary tumors of ER+ breast cancer patients that were either HER2+ or HER2−. Figure 1a shows the basal phosphorylation of 104 peptides (kinase substrates) in 29 primary breast tumors. Based on the level of phosphorylation of the kinase substrates, reflecting the kinase activity, we identified three clusters of peptides through unsupervised clustering, showing different levels of kinase activity. Peptides in cluster 1 exhibited overall very high levels of phosphorylation, whereas peptides in clusters 2 and 3 displayed moderate and low levels, respectively (see supplementary file 4 for information of peptide clusters). The unsupervised clustering distributed the patients in two main groups; one group with lower peptide phosphorylation in each of the clusters identified, and a second group with higher phosphorylation (Fig. 1a). Interestingly, the patients identified at the cluster with lower phosphorylation, were more likely to be PR+ compared to patients identified at peptide clusters with higher phosphorylation peptides (9 out of 11 (~ 82%) at lower vs. 13 out of 18 (~ 72%) at higher phosphorylation, *p* = 3.39E−06). Next, we sought for differences in peptide phosphorylation associated with HER2 and PR expression. In this dataset, we found no statistically significant differences in the level of phosphorylation in overall kinase activity between differential HER2 and PR status in ER+ tumors, nor within the three clusters of peptides identified (see supplementary file 5). Although not significant, we did observe a trend of increased phosphorylation in PR- samples compared to PR+ samples.

To further investigate the kinase activity patterns, we performed in parallel PTK profiling in the well-established ER+ cell lines, MCF7, ZR75-1 and BT474. Western blot of HER2, ER and PGR was performed, showing that they were representative of the human breast tumors with different levels of proteins of HER2 and PR (MCF7 as ER+/HER2−/PR+, BT474 as ER+/HER2+/PR+ and ZR751 as ER+/HER2+/PR-, supplementary file 2). Next, PTK profiling was performed on all the cell lines, which were either untreated (E0) or exposed to estrogen (17β- estradiol) for 10 min (E10) (Fig. 1b). The use of estradiol aimed to test whether estrogen signaling might influence the kinase activity. Our findings indicated that treating cell lines with estradiol did not have a significant impact on the overall activity of PTKs in HER2+ cell lines (ZR75-1 and BT474). However, in the HER2− cell line (MCF7), we found a significant increase of peptide phosphorylation in response to estrogen treatment (supplementary file 6). Furthermore, the analysis revealed that the ZR75-1 cell line, which is a PR- cell line, exhibited increased kinase activity in contrast to the two other cell lines, MCF7 and BT474. The kinase substrates that exhibited higher phosphorylation in ZR75-1 were overlapping with those exhibiting high phosphorylation in cluster 1 and some in cluster 2 from the patient sample data from Fig. 1a. These results motivated us to investigate further the role of PR in tumor tissue.

### PR expression with great influence on phosphorylation in ER+/HER2− tumors

**Fig. 2:**
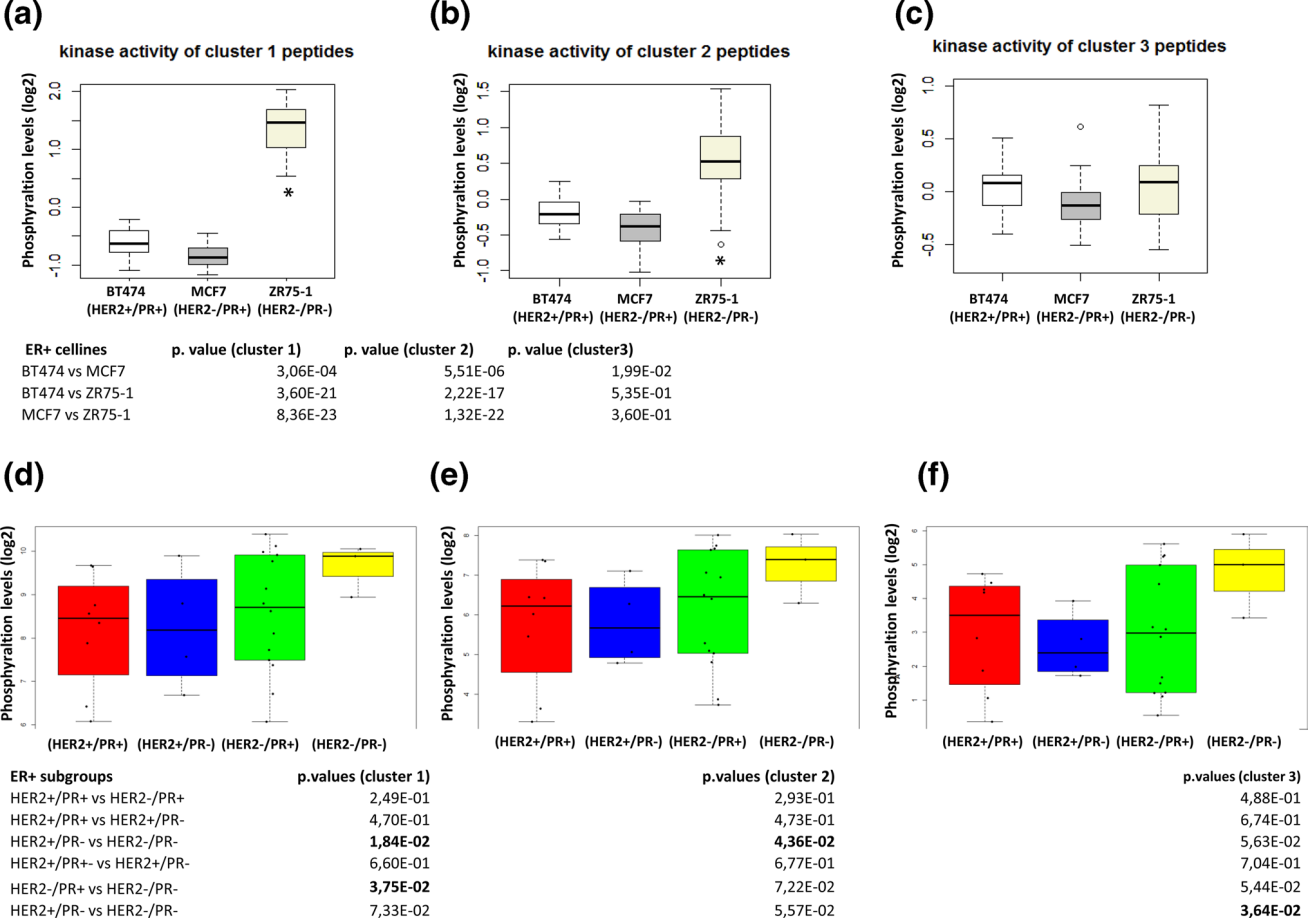
Boxplots of mean phosphorylation of peptides in cluster 1, 2, and 3 in three different ER+ cell lines and **a–c** and different subgroups of ER+ breast cancer samples (**d–f**). The three peptide clusters are the same as the peptide clusters obtained from unsupervised clustering in Fig. 1a. Statistical significance between subgroups/cell lines in each cluster is indicated by *p *values < 0.05 in tables*.* *ER−* estrogen receptor, *PR−* progesterone receptor, *HER2−* human epidermal growth factor receptor 2

To further investigate the correlation between PR and PTKs, we analyzed the average kinase activity in different cell lines (Fig. 2a, b and c) and patient tumor subgroups based on the PR and HER2 status in each of the three peptide clusters (Fig. 2d, e and f). Especially in cluster 1 and cluster 2 peptides, the overall PTK activity levels were significantly increased in the PR- cell line, ZR75-1. In breast tumors, we clearly identified that the subgroup ER+/HER2−/PR- showed overall higher kinase activity compared to the other PR+ subgroups in all three peptide clusters identified through unsupervised clustering in Fig. 1a. Through statistical testing, a total of 30 kinase substrates, representing 26 different PTKs, were identified as significantly differently phosphorylated in ER+/HER2− tumors according to PR status (Table 1). We did not find any differences in phosphorylated peptides when the same analysis was performed with ER+/HER2+ breast cancer samples (data not shown).

**Table 1:** Kinase substrates (p < 0.05) showing difference in activity in PR+ and PR− in ER+/HER2− breast tumors

Substrate ID	Gene symbol	Gene name	*p* (raw)	q*	PR − mean	PR+ mean	LFC
41_654_666	EPB41	Erythrocyte membrane protein band 4.1	2.7E−03	1.7E−03	9.68	8.82	− 0.85
B3AT_39_51	SLC4A1	Solute carrier family 4, anion exchanger, member 1	4.2E−03	3.3E−03	5.34	2.58	− 2.76
CD3Z_146_158	CD247	CD247 molecule	6.9E−03	5.0E−03	7.02	5.19	− 1.83
CDK2_8_20	CDK2	Cyclin-dependent kinase 2	7.2E−03	6.7E−03	10.99	9.96	− 1.02
CRK_214_226	CRK	v-crk sarcoma virus CT10 Oncogene homolog	8.8E−03	8.3E−03	6.02	3.24	− 2.78
EGFR_1165_1177	EGFR	Epidermal growth factor receptor	1.5E−02	1.0E−02	5.90	4.56	− 1.34
EPHA1_774_786	EPHA1	EPH receptor A1	1.6E−02	1.2E−02	9.87	8.82	− 1.06
EPHA2_765_777	EPHA2	EPH receptor A2	1.9E−02	1.3E−02	10.25	9.12	− 1.13
EPHA7_607_619	EPHA7	EPH receptor A7	2.1E−02	1.5E−02	9.15	8.11	− 1.04
EPHB1_771_783	EPHB1	EPH receptor B1	2.2E−02	1.7E−02	9.13	7.97	− 1.16
EPHB1_921_933	EPHB1	EPH receptor B1	2.2E−02	1.8E−02	6.53	3.80	− 2.73
EPOR_361_373	EPOR	Erythropoietin receptor	2.2E−02	2.0E−02	9.21	8.19	− 1.02
EPOR_419_431	EPOR	Erythropoietin receptor	2.6E−02	2.2E−02	8.49	6.87	− 1.62
ERBB2_1241_1253	ERBB2	v-erb-b2 erythroblastic leukemia viral oncogene homolog 2	2.7E−02	2.3E−02	6.76	5.22	− 1.54
ERBB2_870_882	ERBB2	v-erb-b2 erythroblastic leukemia viral oncogene homolog 2	3.1E−02	2.5E−02	6.83	5.12	− 1.70
FES_706_718	FES	Feline sarcoma oncogene	3.1E−02	2.7E−02	10.26	9.18	-1.08
FGFR3_753_765	FGFR3	Fibroblast growth factor receptor 3	3.3E−02	2.8E−02	7.73	6.23	− 1.50
INSR_992_1004	INSR	Insulin receptor	3.3E−02	3.0E−02	5.29	3.08	− 2.22
JAK2_563_577	JAK2	Janus kinase 2	3.5E−02	3.2E−02	7.79	6.68	− 1.12
LCK_387_399	LCK	Lymphocyte-specific protein tyrosine kinase	3.7E−02	3.3E−02	8.68	7.61	− 1.07
MK14_173_185	MAPK14	Mitogen-activated protein kinase 14	3.8E−02	3.5E−02	5.11	3.17	− 1.94
ODBA_340_352	BCKDHA	Branched chain keto acid dehydrogenase E1. alpha polypeptide	4.0E−02	3.7E−02	5.78	2.93	− 2.85
P85A_600_612	PIK3R1	Phosphoinositide-3-kinase, regulatory subunit 1 (alpha)	4.3E−02	3.8E−02	10.50	9.17	− 1.32
PAXI_24_36	PXN	Paxillin	4.3E−02	4.0E−02	10.38	9.02	− 1.37
PDPK1_369_381	PDPK1	3-phosphoinositide-dependent protein kinase-1	4.6E−02	4.2E−02	8.52	7.75	− 0.77
RET_1022_1034	RET	Ret proto-oncogene	4.7E−02	4.3E−02	9.82	8.84	− 0.98
STAT4_714_726	STAT4	Signal transducer and activator of transcription 4	4.7E−02	4.5E−02	6.05	3.90	− 2.15
VGFR1_1040_1052	FLT1	FMS-related tyrosine kinase-1	4.7E−02	4.7E−02	5.54	3.62	− 1.92
VGFR2_1046_1058	KDR	Kinase insert domain receptor	4.8E−02	4.8E−02	5.17	2.71	− 2.46
VGFR2_989_1001	KDR	Kinase insert domain receptor	4.8E−02	5.0E−02	9.53	8.48	− 1.05

*LFC* log fold change, *P* p.value, *PR* progesterone receptor

^*^Corrected *p *value

**Fig. 3:**
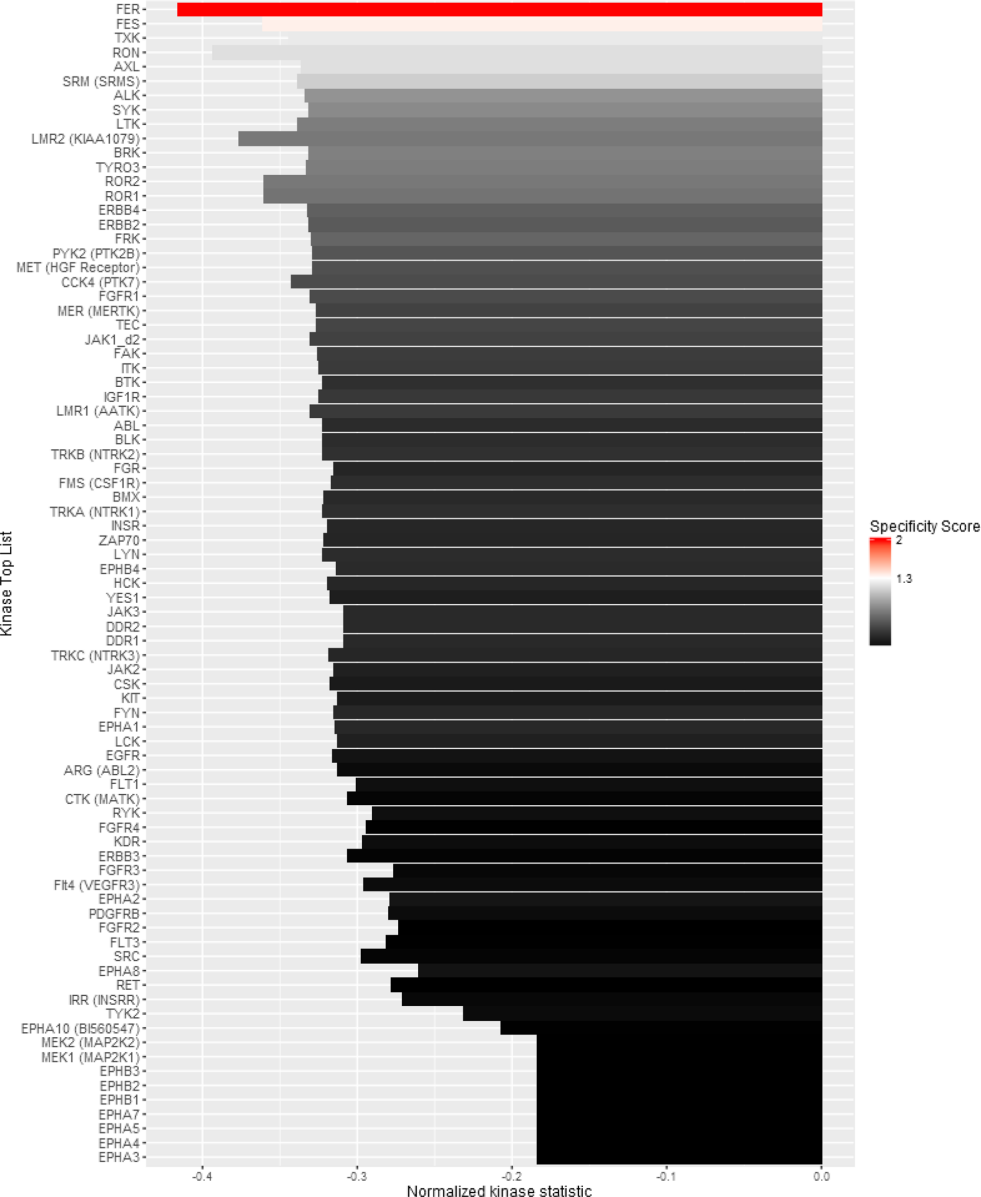
Upstream kinases responsible for changes in ER+/HER2− tumors with differential PR status. There are 75 putatively affected kinases (*y *axis) in ER+/HER2− tumors that are negatively expressed in PR+ (i.e., positive expressed in PR−). Length of the bars indicates the value of *τ* for each of these kinases. (*τ* > 0 indicates lower activity in PR+ samples compared to PR− samples). The color of the bars indicates the specificity score. Positive score means higher expressed, whereas negative score indicates low expression of the putative kinases. The kinases on the top are more likely to be upstream, compared to the kinases at the bottom of the list

In our analysis, PR- tumors showed higher kinase activity of these 30 kinase substrates compared to PR+ tumors within the ER+/HER2− subgroup, as reflected by higher mean phosphorylation values. Several membrane residing PTK receptors showed higher phosphorylation in ER+/HER2−/PR- tumors, including HER2, insulin receptor (INSR), vascular endothelial growth factor receptor (VEGFR), fibroblast growth factor receptor (FGFR), erythropoietin receptor (EPOR) and Erythropoietin-Producing Hepatoma Receptor (EPHA). Through pathway analysis [25, 26], we identified several of the PTKs to be part of the phosphoinositide-3-kinase/Akt (PI3K) signaling pathway (*p *value 4.10E−08, benjamini 4.47E−06), Ras signaling pathway (*p *value 1.47E−07, benjamini 5.33E−06) VEGF pathway (*p *value 9.61E−05, benjamini 7.48E−04) and ErbB pathway (*p* value 5.03E−03, benjamini 2.58E−02). In order to identify the kinases that might be responsible for the differential phosphorylation of kinase substrates in ER+/HER2− tumors with differential PR status, we performed upstream kinase analysis as described above. In total, 75 kinases were identified, with the ones on the top of the table being more likely to be responsible for the changes in kinase phosphorylation levels between PR+ and PR− in ER+/HER2− tumors (Fig. 3). In agreement with the previous pathway analysis, RAS signaling (*p* value 6.9E−8, benjamini 2.2E−6), PI3K signaling (*p* value 5.3E−06, benjamini 1.0E−04) and ErbB signaling pathways (*p* value 1.6E−5, benjamini 2.5E−4) were identified as the top pathways to be involved in the difference of upstream kinases between PR− and PR+ in ER+/HER2− tumors. Altogether, our findings so far suggest that the loss of PR leads to increased kinase activation in ER+/HER2−/PR− tumors, involving the PI3K pathway.

### mRNA expression differs in ER+/HER2− breast tumors based on PR status

**Fig. 4:**
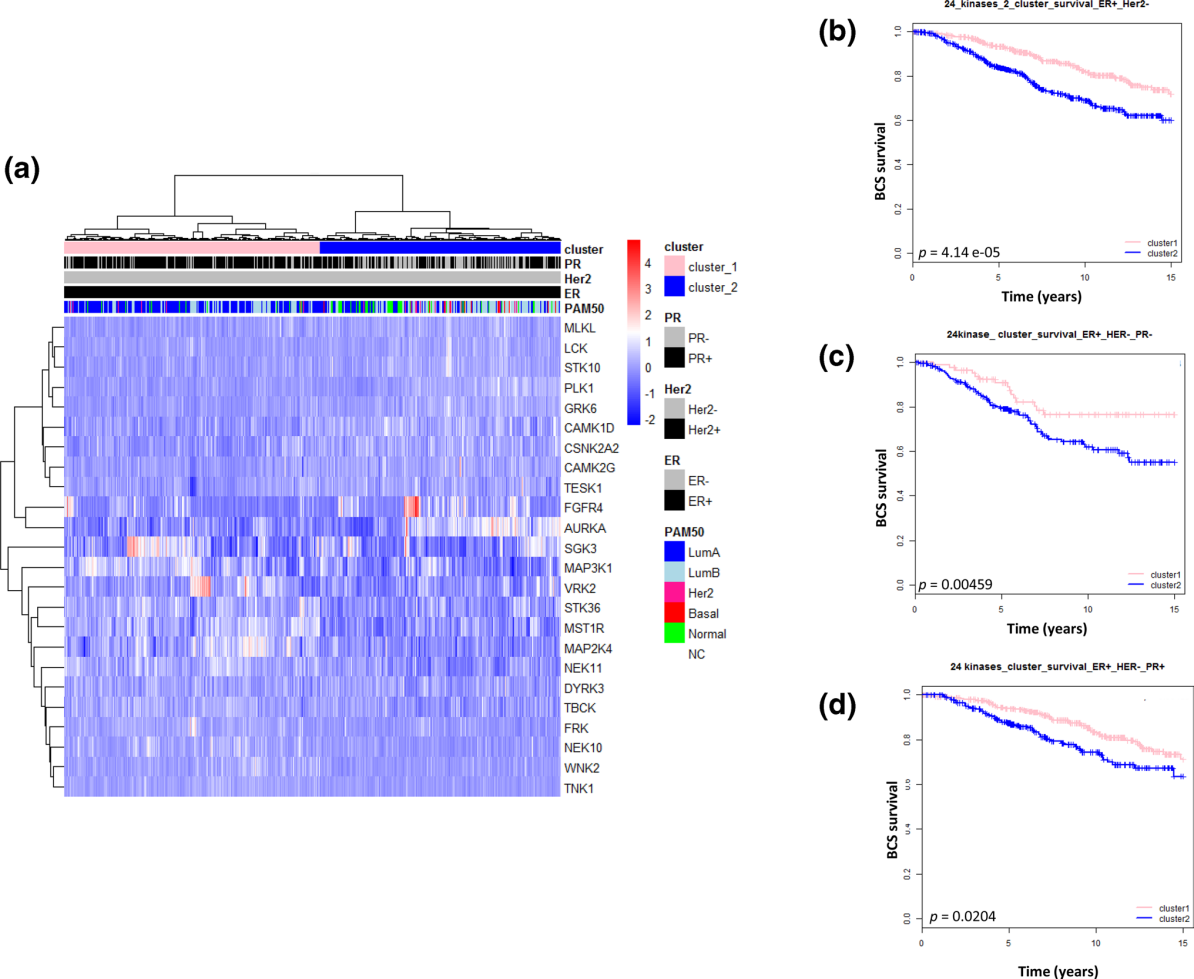
**a** Heatmap with clustering of 24 kinase-encoding genes that are differentially expressed in ER+/HER2− tumors with differential PR status. Red on the heatmap indicates higher expression levels, whereas blue indicates lower mRNA expression levels. Survival analysis of breast tumors grouped by gene expression in cluster 1 (pink) and cluster 2 (blue) are shown as Kaplan–Meier survival plots of patients whose tumors were grouped into **b** ER+/HER2−, **c** ER+/HER2−/PR−, and **d** ER+/HER2−/PR+. *ER *estrogen receptor, *PR *progesterone receptor, *Her2 *human epidermal growth factor 2

Our data have so far suggested that the loss of PR expression might ameliorate the activation of kinases in ER+/HER2− tumors and cell lines. In order to explore further the relation of PR expression in HER2− tumors, we exploited the mRNA expression dataset from the METABRIC cohort [23], using data from only ER+ tumors (*n* = 1052). Our analysis revealed a set of 648 genes that were identified as significantly differentially expressed (Bonferroni corrected *p* value < 0.05) between PR+ and PR− in HER2− tumors (*n* = 977) (supplementary file 7), with most of the genes being involved in pathways related to tyrosine kinase activity, and also immunity (supplementary file 8). Survival analysis with all 648 genes revealed that patients that lack PR in ER+ tumors, had significantly worse survival than patients that were PR+ (see supplementary file 10). Again, no significant differences in gene expression based on PR status in ER+/HER2+ breast tumor samples were observed, except the expression of the PGR gene itself (see Supplementary file 9). These findings are in agreement with our kinase activity data. Among the differentially expressed genes (*n* = 648) identified between PR+ vs*.* PR− in ER+/HER2− tumors, we identified 24 genes that encode for known human PTKs obtained from previous reports [13]. Moreover, we performed a heatmap with hierarchical clustering using the 24 kinase-encoding genes (bonferroni corrected *p *value < 0.05) across ER+/HER2− breast cancer samples. The analysis clearly divided the patients in two clusters (cluster 1 and cluster 2) (Fig. 4a). The majority of the PR− tumor samples were grouped together in cluster 2, whereas the majority of PR+ tumor samples were grouped together in cluster 1, with some exceptions. Through survival analysis, we observed that patients in cluster 2 with ER+/HER2− tumors have significantly worse survival compared to patients in cluster 1 (Fig. 4b). Furthermore, patients in cluster 2, which predominantly include PR- patients, have a significantly worse survival than PR- patients in cluster 1 (Fig. 4c). Even more interesting, PR+ patients seem to have a significant worse survival in cluster 2 than PR+ patients in cluster 1 (Fig. 4d). Hence, the expression of PR seems to play a role in survival when considering these two clusters and the expression of the 24 kinase genes analyzed in the present study.

### PR regulation through protein tyrosine kinases

**Fig. 5:**
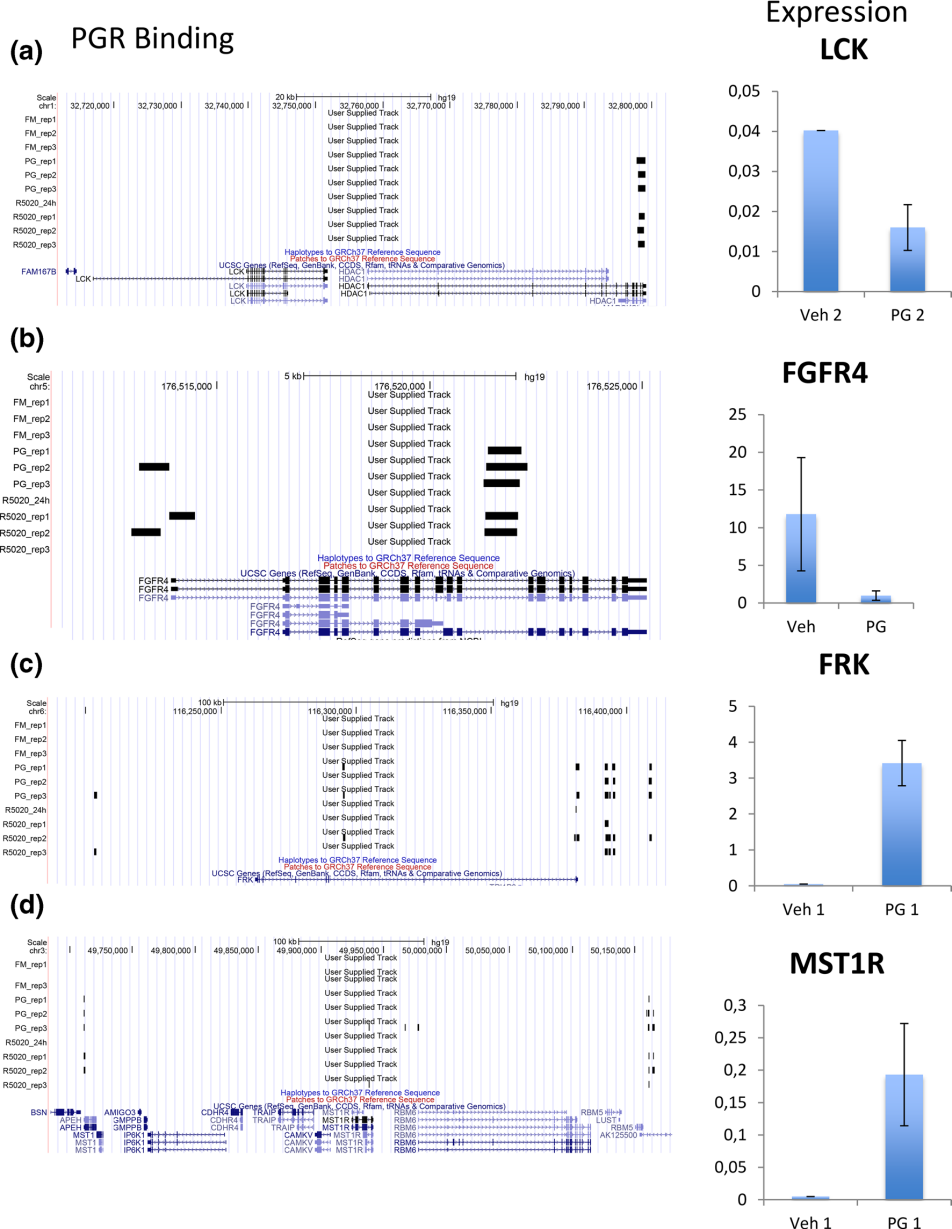
PGR binding sites and RNA expression in **a** LCK, **b** FGFR4, **c** FRK, and **d** MST1R through PR chip sequencing and RT-PCR in untreated MCF7 cells (veh) and in cells treated with progesterone (PG2)

Next, we found out that four kinases were part of this 24 gene list that were also identified in the upstream kinase analysis previously. These four kinase genes were, fyn-related Src family tyrosine kinase (FRK), fibroblast growth factor receptor 4 (FGFR4), Lymphocyte Cell-Specific Protein Tyrosine Kinase (LCK), and Macrophage Stimulating 1 Receptor (MST1R). They were all identified as significantly differentially expressed between PR+ and PR− in ER+/HER2− tumors (bonferroni *p* value < 0.05), and all involved in the PI3K pathway [29–32]. When analyzing the expression of these genes in cluster 1 and cluster 2, we identified that FGFR4 and LCK exhibit significantly higher expression in cluster 2, whereas FRK and MST1R show lower expression in cluster 2 compared to cluster 1 (supplementary file 11a). Furthermore, FGFR4 and LCK showed higher expression in PR− tumors, whereas FRK and MST1R show lower expression in PR- tumors. This finding was validated with RT-PCR on ER+/HER2− breast tumor samples (see supplementary file 11b), except for MST1R, which showed no difference between PR+ and PR-. Furthermore, in ER+/HER2+ tumors, we did not observe the same trend of expression for these four kinases (supplementary file 11c). In ER+/HER2+ tumors, it seems like at least for LCK and FGFR4, their expression is opposite to what is observed in ER+/HER2− tumors. In ER+/HER2+ tumors, FGFR4 and LCK have much lower levels of FGFR4 and LCK in PR− compared to PR+ tumors. To validate our findings experimentally, we determined whether PR regulates the gene expression of the four identified kinases (FGFR4, LCK, FRK and MST1R) in ER+/HER2−/PR+ cells (MCF7). For this purpose, we first determined the binding of PR at chromatin regions from publicly available ChIP-sequencing data of these four kinases investigated [24]. We identified a gain of PR binding in cells stimulated with progesterone receptor agonists. Furthermore, we analyzed the RNA expression of the four kinases in breast cancer cells stimulated with progesterone. Briefly, breast cancer cells were plated and hormone-deprived for three consecutive days and either treated with ethanol (vehicle) or treated with progesterone (PG2). Then, the gene expression of FGR4, LCK, FRK and MST1R was determined by RT-PCR and compared the expression of cells stimulated with progesterone vs control cells. Our in vitro experiments validated that the gene expression of LCK and FGFR4 was downregulated by progesterone (Fig. 5a, b), whereas for FRK and MST1R, the expression was increased (Fig. 5c, d), which is in concordance with the findings in clinical samples. We also determined the binding of PR at chromatin regions of these four kinases investigated, and we identified a gain of PR binding in cells stimulated with progesterone. All these findings suggest that PR controls the activity of several pivotal kinases in ER+/HER2− patients.

## Discussion

Since the clinical success of HER2-targeted therapies, like trastuzumab and pertuzumab, there has been an increased interest in PTKs as potentially attractive intracellular targets for breast cancer treatment. While HER2 has a profound impact on breast cancer biology and prognosis, one might expect that differences in HER2 status alone would have a significant effect on the activity of downstream kinases in ER+ breast cancers. However, when analyzing the phosphorylation patterns of kinases in ER+ breast cancer samples and cell lines, we observed no significant differences in phosphorylation of kinase substrates that could be correlated to HER2 status. However, it is important to mention that activation of HER2 may occur through several mechanisms in breast cancer [33]. For instance, it has been reported that there are rare types of lung cancers that have HER2 kinase domain mutations that confer increased kinase activity without overexpression [33]. This could potentially explain why we did not observe any differences between HER2+ and HER2− in our dataset, at least for the analysis involving phosphorylation of kinase substrates.

The role of estrogens and ER in breast cancer is undisputed, and drugs inhibiting estrogen synthesis or ER itself are effective cancer therapies for HR-positive tumors. However, the action of PR in breast cancer is still somehow underexplored and remains controversial. PR expression is induced by the activation of ER, and PR-related signaling pathways have important roles in the induction, progression and maintenance of neoplastic phenotype in breast cancer [34]. Previous research also shows that PR is not merely an ERα-induced gene target, but is also an ERα-associated protein that modulates its behavior by controlling chromatin binding and transcriptional activity, which has important implications for prognosis and therapeutic interventions [24]. Although it seems like PR appears to have a profound effect on prognosis and aggressiveness of breast cancer, the kinases and pathways that come to play is less studied.

In this study, we identified PR as a plausible factor responsible for differences in kinase activity within ER+ tumors. Higher kinase activity was observed in ER+/HER2−/PR− tumors compared to tumors that are positive for PR expression. Furthermore, using gene expression data from the METABRIC cohort [23], we revealed that the loss of PR expression had a pronounced effect on survival in ER+ patients in general, but the differences were even more significant in ER+/HER2− patients, even when including a 24 kinase-encoding gene signature only. Using the 24-kinase-encoding-gene signature, we identified two clusters with differences in survival in ER+/HER2− patients. Cluster 1, which consisted mostly of PR+ patients, exhibited better survival than patients in cluster 2, which were mostly PR-. Interestingly, the patients within cluster 2 that were PR+ had worse survival compared to patients with same clinical features in cluster 1, indicating that the expression of kinase-encoding genes themselves might indicate worse survival altogether. Our data are so far in agreement with previous studies, which have shown that clinically, the PR status exerts a significant impact on the prognosis of ER+ breast cancer patients in which patients with PR- tumors have a worse outcome than PR+ tumors [35–37]. Prat et al. [38] also reported that a cut-off value of > 20% PR tumor cell positivity is a significant prognostic factor within luminal-type breast cancers. Also, the extent of PR expression has shown to be a potent prognostic indicator that can aid evaluations of the long-term prognosis of ER+/HER2− breast cancers [34]. However, in our study, probably due to the small sample set, we could not observe differences in phosphorylation in patients with high or low PR tumor cell positivity, and as for the gene expression data, such differences in PR levels were not available. One might speculate that the PR+ patients that clustered together with patients that were PR- and vice versa could be due to differences of the amount of PR− positive tumor cells.

Furthermore, we identified four kinases that were important for the differences between PR+ and PR- in ER+/HER2− tumors, namely FGFR4, LCK, FRK and MST1R. These four kinases were identified through upstream analysis as potential kinases that might be responsible for changes between PR+ and PR− in ER+/HER2− tumors, but they were also identified in the METABRIC gene expression dataset as significantly differentially expressed between these two groups. Interestingly, we could not find any significant genes (except PGR) or kinases that were different between PR+ and PR− in ER+/HER2+ tumors. One might postulate that in ER+/HER2+ tumors, the HER2 pathway may be the major driver for tumor growth and hence there are less obvious differences observed between PR+ and PR- tumors.

FGFR4 and LCK had higher expression in HER2−/PR− samples, whereas FRK and MST1R had lower expression in HER2−/PR− samples. This was validated in breast tumor tissue through RT-PCR, except for MST1R. We also identified these kinases to be regulated directly by PR itself through PR chip-sequencing data. By adding progesterone to MCF7 cells (PR+), we observed that both FGFR4 and LCK were reduced in expression, whereas FRK and MST1R were increased. This is in concordance with our study where we see the complete opposite happening in PR- tumors. These findings are in the agreement of the idea that PR controls the expression of these kinases in ER+/HER2− tumors, and the deregulation of their activity might confer bad prognosis, and be important in treatment.

While PR+ tumors are associated with better prognosis in ER+ breast tumors [4], the loss of PR expression is associated with differential breast tumor responses to anti-ER therapies [7–9]. However, the role of PR activation seems to be a bit controversial, because in some studies, PR activation seems to promote breast cancer, whereas in some studies, progesterone treatment has been shown to be anti-proliferative in ER+/PR+ breast cells [39–41]. At the same time, previous studies have shown that PR itself can be phosphorylated and activated independent of progesterone, through different growth factors and pathways, in particular through MAPK and PI3K/AKT pathway [42]. Experimental data have even implied that growth factor signaling mediates PR downregulation through the activation of the PI3K–Akt–mammalian target of rapamycin (mTOR) pathway [7].

Because PR is regulated by estrogen through ER, the ER-mediated signaling is important in ER+/PR+ tumors. However, in ER+/PR− tumors, one might hypothesize that they rely on other signal transduction pathways to grow because ER-mediated signaling is less important in these tumors. Therefore, the findings that more kinases were identified as differently expressed between PR+ and PR- in ER+/HER2− cancer is perhaps not completely unexpected. However, ER+/PR- cell lines are still dependent of ER for tumor growth. If ER is depleted in these cell lines, then the tumor growth is inhibited. The same happens with hormone resistant cell lines (most of them are PR-). In the case of ER+/PR− patients, they are less sensitive to hormone therapy but still responsive [37]. The findings of the work in this study reveal that the loss of PR function leads to an increase of activity (and in some cases increase of expression as well) of kinases (different to HER2). This effect is not influenced by estrogen-ER activation. Hence, we might believe that the action is dependent of PR. However, we also know that ER, PR, and HER2 are related to each other, and gene signaling occur through a loop of network, which make further studies a necessity to understand their importance in cancer.

The results obtained in this study clearly demonstrated that the Ras/MAPK and PI3K pathways are important, and highly active in ER+/HER2− tumors lacking PR. When analyzing gene expression data, the PI3K cascade does not come up as significant between ER+/HER2− tumors with differential PR status, although, tyrosine kinase activity and immune pathways seems to be much more prominent. The activation of the PI3K pathway and its downstream targets is rather complex. Especially breast cancer tumorigenesis is believed to depend on the PI3K pathway because the majority of breast cancer cases harbor at least one mutation that potentially affects this pathway [34]. Furthermore, the PI3K pathway is commonly altered in ER+/HER2− and ER+/PR− cancers [37, 43]. The PI3K pathway is activated through phosphorylation of several proteins, among them also the four tyrosine kinases that we have identified in this study.

MST1R encodes for the protein RON, which is a RTK for the ligand macrophage-stimulating protein (MSP, MST1). Aberrant expression of RON is associated with tumor progression in breast cancer through its involvement with PI3K, MAPK, JNK, β-catenin, and STAT pathways [44]. Overexpression of RON is associated with cell dissociation, motility and matrix invasion, which all are surrogate markers of an aggressive cancer phenotype with metastatic potential [45]. In our study, the MST1R gene was lower expressed in PR− vs PR+ in ER+/HER2− in the METABRIC dataset, but could not be validated. Since this gene is a marker of invasion, and all our samples were primary tumors, this could possibly explain the reason why we could not see difference in expression, and it could most likely be considered as a marker of late stage disease.

FRK interacts and phosphorylate the tumor suppressor protein PTEN, and through its activation inhibits PI3K/AKT signaling pathway [29]. Low levels of FRK show increased cell growth, colony formation and tumor growth, whereas high levels suppress tumor growth in breast cancer cell lines [46]. In our study, FRK is lower expressed in PR− tumors compared to PR+ in ER+/HER2− tumors, making it an interesting marker for prognosis and potentially drug interventions.

Phosphorylated FGFR4 recruits and phosphorylates two important intracellular targets, phospholipase γ (PLCγ) and FGFR substrate 2 (FRS2). The MAPK and PI3K/AKT pathway can then be triggered by activated FRS2 through recruitment of growth factor receptor bound 2 (GRB2). Previous research has shown that high levels of FGFR4 mRNA could be an independent predictive factor, as high levels of FGFR4 show shorter progression-free survival in breast cancer patients treated with tamoxifen [30, 47]. The FGFR4 expression levels also seem to be significantly increased in doxorubicin-resistant breast cancer clones [48].

LCK itself is mostly found in T cells, and is involved in recruitment and activation of proteins, such as PLC and PI3K, that in turns activates the MAPK and PI3K/AKT pathway [31]. The fact that genes involved in immunity were prominent in PR- compared to PR+ in ER+/HER2− tumors, makes LCK an important target, as tumor infiltrating lymphocytes (TILs) are important components of the microenvironment of the cancer. In breast cancer, they are associated with clinical outcomes as high TIL score is associated with worse prognosis and they provide prognostic information and can predict the patient’s response to neoadjuvant chemotherapy [32]. In our study, both FGFR4 and LCK were found to be higher expressed in HER2−/PR− tumors vs HER2−/PR+. In HER2+ tumors, LCK and FGFR4 seems to be downregulated rather than upregulated in PR- tumors, which is the opposite to what we see in HER2− tumors. The fact that this overexpression is only observed in ER+/HER2− samples and not in ER+/HER2+ samples, seems to agree with what we find, that HER2− tumors are indeed much different from HER2+ tumors, and that HER2−/PR− are more “aggressive” or “oncogenic” in that sense. It could also mean that HER2 together with PR regulate their expression. One could postulate that when HER2 is present, HER2 could be more dominant than PR, and thereby the oncogenic events might occur through different genes/pathways. However, when HER2 is missing, other genes and pathways are more prominent.

As several studies have pointed out the importance of the PI3K pathway during resistance to endocrine therapy in breast cancer patients (39), together with the fact that ER+/PR+ breast cancer responds better to endocrine therapy compared to ER+/PR- breast cancer [35–37], makes it of uttermost importance to study the consequences of the loss of PR in ER-positive breast cancers. The kinases LCK, FRK, FGFR4 and MST1R, all interacting with proteins within the PI3K pathway, seem to be a good starting point according to our findings.

## Conclusions

Taken together, our results suggest that loss of PR in ER+ breast cancer influences significantly the downstream tyrosine kinase signaling, possibly through multiple mechanisms and involving several kinases linked to the PI3K pathway.

## Electronic supplementary material

Below is the link to the electronic supplementary material.

Supplementary file1 (PDF 208 kb)

Supplementary file2 (PDF 17 kb)

Supplementary file3 (PDF 166 kb)

Supplementary file4 (PDF 391 kb)

Supplementary file5 (PDF 14 kb)

Supplementary file6 (PDF 686 kb)

Supplementary file7 (XLSX 113 kb)

Supplementary file8 (PDF 203 kb)

Supplementary file9 (XLSX 1705 kb)

Supplementary file10 (PDF 173 kb)

Supplementary file11 (PDF 198 kb)
