# Supplementary material for: Loss of progesterone receptor is associated with distinct tyrosine kinase profiles in breast cancer
Source: Breast Cancer Res Treat. 2020 Jul 24;183(3):585–98. doi: 10.1007/s10549-020-05763-7 (PMC7497693; doi:10.1007/s10549-020-05763-7)
Supplement: Supplementary file 1 — Supplementary file1 (PDF 208 kb) [file 10549_2020_5763_MOESM1_ESM.pdf]

Sample ID	Gender	Histology	Age at op.	ER status	PR status	Her2 status	Ki-67 %	Ki-67% Hotspot	Grade	T	N
CM549T	Female	muc adeno	89	>50	>50	-	27-21	21	II	2	0
CM561T	Female	IDC	67	>50	>10	-	20-24	24	II	1b	0 (15)
CM575T	Female	IDC	78	>50	>10	-	15-38	38	III	2	1 (3/16)
CM576T	Female	muc ca	73	>50	>50	-	15-24	24	II	2	0
CM609T	Female	IDC	48	100	-	-	>50	50	II	2	0
CM615T	Female	IDC	80	-	>10	3+	28-45	45	III	1c	1 1/12
CM617T	Female	IDC	79	>50	>10	-	6-10	10	I	2	1 1/15
CM619T	Female	IDC	71	>50	>10	-	28-38	38	II	2	0
CM623T	Female	IDC	48	90	90	3+	26-43	43	III	2	1 1/16
CM624T	Female	ILC	65	90	90	3+ (amp)	55-70	70	III	2	0
CM630T	Female	IDC	67	>50	>10	-	40-55	55	III	2	0
CM636T	Male	IDC	71	>50	>10	-	38-54	54	III	2	1 1 /15
CM638T	Female	IDC	71	>50	>10	-	5-15	15	II	2	2 9/9
CM641T	Female	IDC	91	-	-	3+	24-42	42	III	2	1 1/17
CM651T	Female	IDC	78	100	40	3+	19-33	33	III	2	1 2/16
CM656T	Female	medular ca IDC	37	~1-10	-	-	80	80	III	2	0
CM657T	Female	IDC	77	90-100	>50	3+	18-25	25	II	2	1 1/7
CM659T	Female	IDC	63	>50	>10	-	~10-15	15	II	2	1 1/11
CM712T	Female	IDC	62	>50	-	3+	40-51	51	III	1c	2 4/10
CM713T	Female	IDC	56	>10	-	3+ (amp)	38-54	54	III	2	0
CM714T	Female	IDC	44	90	>90	3+	44-48	48	III	2	0
CM733T	Female	IDC	45	100	100	-	23	23	II	1c	0
CM740T	Female	IDC	49	-	-	+	60-80	80	III	2	0
CM741T	Female	IDC	67	>80	>50	3+	20-25	25	II	1b	0
CM744T	Female	IDC	63	100	>10	+ (amp)	23-38	38	III	2	1 1/2
CM748T	Female	IDC	75	>50	-	3+	50-60	60	III	2	0
CM752T	Female	IDC	65	100	50	-	2,3-4,5	4,5	II	1c	0
CM764T	Female	IDC	60	>90	-	-	10-27	27	I	2	0
CM774T	Female	IDC	79	>50	>50	-	17-25	25	II	1c	1 1/3
CM775T	Female	IDC	77	>50	>10	-	6-12	12	II	2	3 10/21
CM779T	Female	IDC	66	>50	>10	3+	32-35	35	III	1c	0
CM782T	Female	Apokrin IDC	54	50	-	+	22-35	35	III	1c	1 2/18

Abbreviations: IDC - intraductal carcinoma, ILC: intralobular carcinoma, op- operation, ER- estrogen receptor, PR- progesterone receptor, T- tumor stage, N- lymphnode spread
