# Supplementary figures and images for: Loss of progesterone receptor is associated with distinct tyrosine kinase profiles in breast cancer
Source: Breast Cancer Res Treat. 2020 Jul 24;183(3):585–98. doi: 10.1007/s10549-020-05763-7 (PMC7497693; doi:10.1007/s10549-020-05763-7)

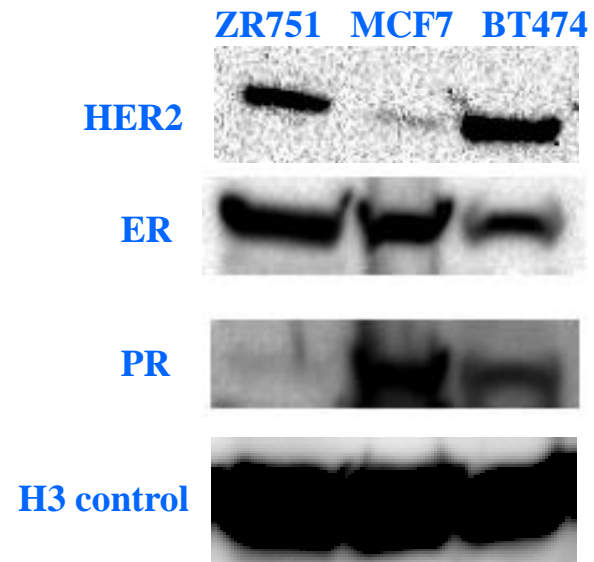

Supplement: Supplementary file 2 — Supplementary file2 (PDF 17 kb) [file 10549_2020_5763_MOESM2_ESM.pdf]

a)

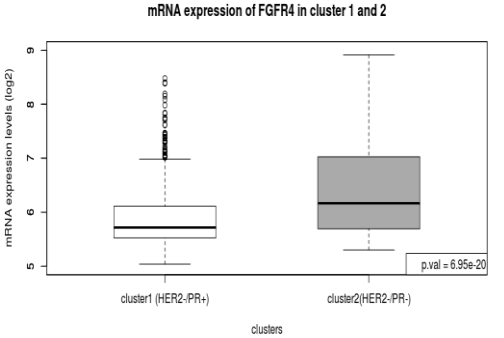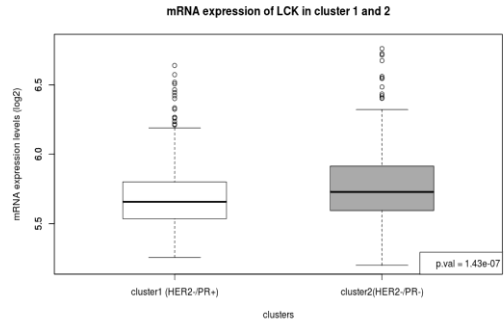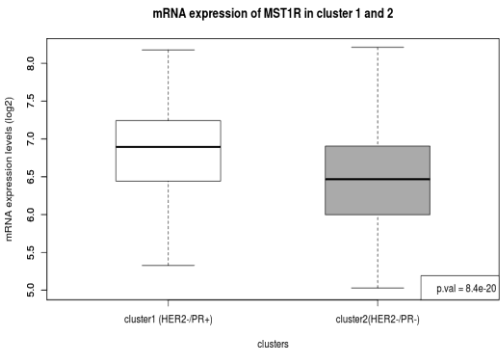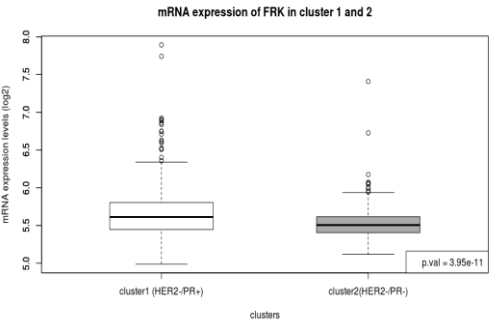

b)

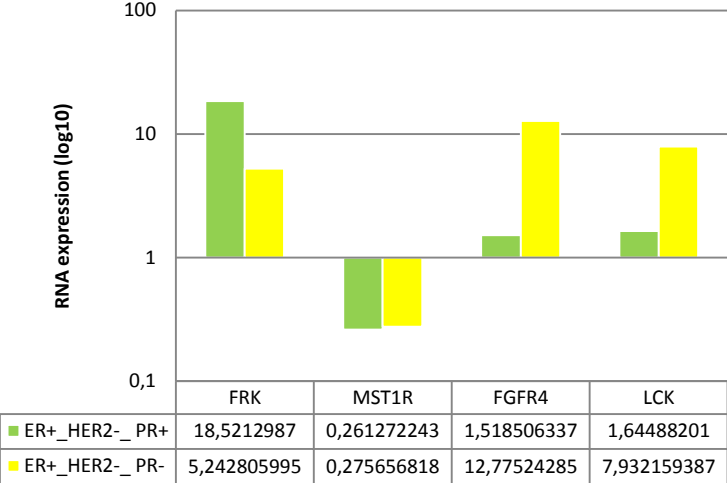

c)

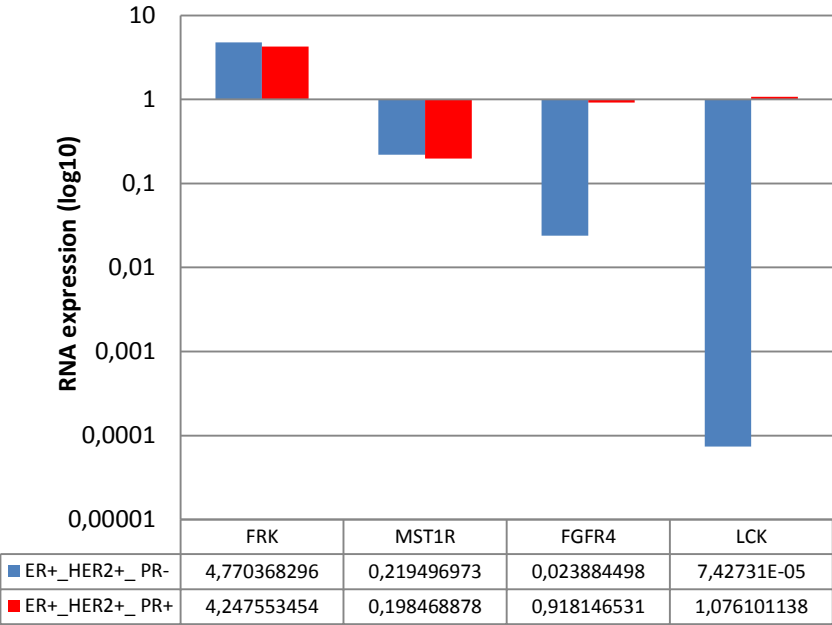

Supplement: Supplementary file 11 — Supplementary file11 (PDF 198 kb) [file 10549_2020_5763_MOESM11_ESM.pdf]
