# Supplementary material for: Loss of progesterone receptor is associated with distinct tyrosine kinase profiles in breast cancer
Source: Breast Cancer Res Treat. 2020 Jul 24;183(3):585–98. doi: 10.1007/s10549-020-05763-7 (PMC7497693; doi:10.1007/s10549-020-05763-7)
Supplement: Supplementary file 3 — Supplementary file3 (PDF 166 kb) [file 10549_2020_5763_MOESM3_ESM.pdf]

Gene	Primer sequence
FRK	FW CGAGCAGGTGACAAACTTCA<br>REV TGCCTGTAGGCTTCTGTCCT
LCK	FW ATCCCTTACCCAGGGATGAC<br>REV TCAAGGCTGAGGCTGGTACT
FGFR4	FW AGCACCCTACTGGACACACC<br>REV ACGCTCTCCATCACGAGACT
MST1R	FW CAAGGTCTGGACGACAACTATTG<br>REV CAGTGGTGGTATTGGCTGTG
ERBB2	FW AAAGGCCCAAGACTCTCTCC<br>REV CAAGTACTCGGGGTTCTCCA
ESR1	FW TGATTGGTCTCGTCTGGCG<br>REV CATGCCCTCTACACATTTTCCC
UBC	FW ATTTGGGTCGCGGTTCTTG<br>REV TGCCTTGACATTCTCGATGGT
TBP	FW GCCAGCTTCGGAGAGTTCTG<br>REV GCACGAAGTGCAATGGTCTTT
