# Supplementary material for: Loss of progesterone receptor is associated with distinct tyrosine kinase profiles in breast cancer
Source: Breast Cancer Res Treat. 2020 Jul 24;183(3):585–98. doi: 10.1007/s10549-020-05763-7 (PMC7497693; doi:10.1007/s10549-020-05763-7)
Supplement: Supplementary file 4 — Supplementary file4 (PDF 391 kb) [file 10549_2020_5763_MOESM4_ESM.pdf]

**peptides in cluster 1**	**peptides in cluster 2**	**peptides in cluster 3**
41_654_666	ACHD_383_395	AMPE_5_17
CBL_693_705	ANXA1_14_26	B3AT_39_51
CDK2_8_20	ANXA2_17_29	C1R_199_211
EPHA1_774_786	CALM_95_107	CRK_214_226
EPHA2_765_777	CD3Z_116_128	DDR1_506_518
EPHA7_607_619	CD3Z_146_158	DYR1A_212_224
EPHB1_771_783	CDK7_157_169	EGFR_1118_1130
EPOR_361_3732	CTNB1_79_91	EPHA4_589_601
FAK2_572_584	DCX_109_121	EPHB1_921_933
FER_707_719	DYR1A_312_324	EPHB4_583_595
FES_706_718	EGFR_1103_1115	ERBB4_1181_1193
LAT_249_261	EGFR_1165_1177	ERBB4_1277_1289
LCK_387_399	EGFR_1190_1202	INSR_1348_1360
NTRK2_696_708	EPOR_419_431	INSR_992_1004
P85A_600_612	ERBB2_1241_1253	MBP_259_271
PAXI_24_36	ERBB2_870_882	MBP_263_275
PDPK1_2_14	FAK1_569_581	MK14_173_185
PDPK1_369_381	FGFR1_761_773	ODBA_340_352
PECA1_706_718	FGFR2_762_774	RBL2_99_111
PGFRB_1014_1028	FGFR3_753_765	STAT4_714_726
RAF1_332_344	JAK1_1015_1027	TNNT1_2_14
RASA1_453_465	JAK2_563_577	VGFR1_1040_1052
RET_1022_1034	K2C6B_53_65	VGFR1_1049_1061
VGFR2_989_1001	K2C8_425_437	VGFR1_1206_1218
ZAP70_485_497	KSYK_518_530	VGFR1_1235_1247
	LAT_194_206	VGFR2_1046_1058
	MBP_198_210	
	MET_1227_1239	
	MK01_180_192	
	MK07_211_223	
	MK10_216_228	
	MK12_178_190	
	NCF1_313_325	
	NPT2A_501_513	
	PGFRB_1002_1014	
	PGFRB_709_721	
	PGFRB_768_780	
	PGFRB_771_783	
	PP2AB_297_309	
	PRGR_786_798	
	PRRX2_202_214	
	PTN11_539_551	
	RB_804_816	
	RON_1346_1358	
	RON_1353_1365	
	TEC_512_524	
	TYRO3_679_691	
	VGFR1_1326_1338	
	VGFR2_1052_1064	
	VGFR2_1168_1180	
	VGFR2_944_956	
	VGFR3_1061_1073	
	VINC_815_827	
