# Supplementary material for: Loss of progesterone receptor is associated with distinct tyrosine kinase profiles in breast cancer
Source: Breast Cancer Res Treat. 2020 Jul 24;183(3):585–98. doi: 10.1007/s10549-020-05763-7 (PMC7497693; doi:10.1007/s10549-020-05763-7)
Supplement: Supplementary file 5 — Supplementary file5 (PDF 14 kb) [file 10549_2020_5763_MOESM5_ESM.pdf]

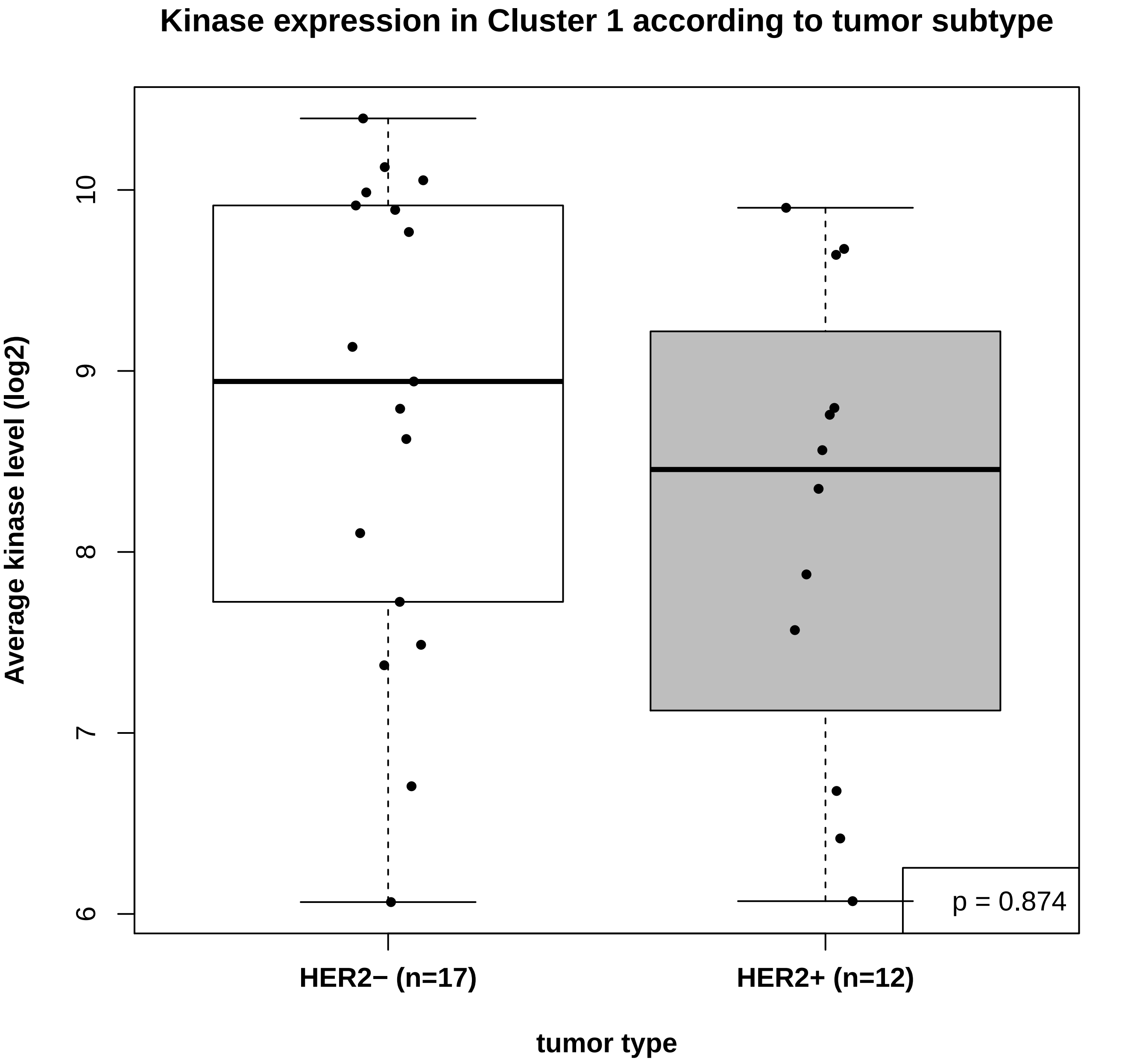
Kinase expression in Cluster 1 according to tumor subtype
Average kinase level (log2)
10
9
8
7
6
p = 0.874
HER2− (n=17)
HER2+ (n=12)
tumor type

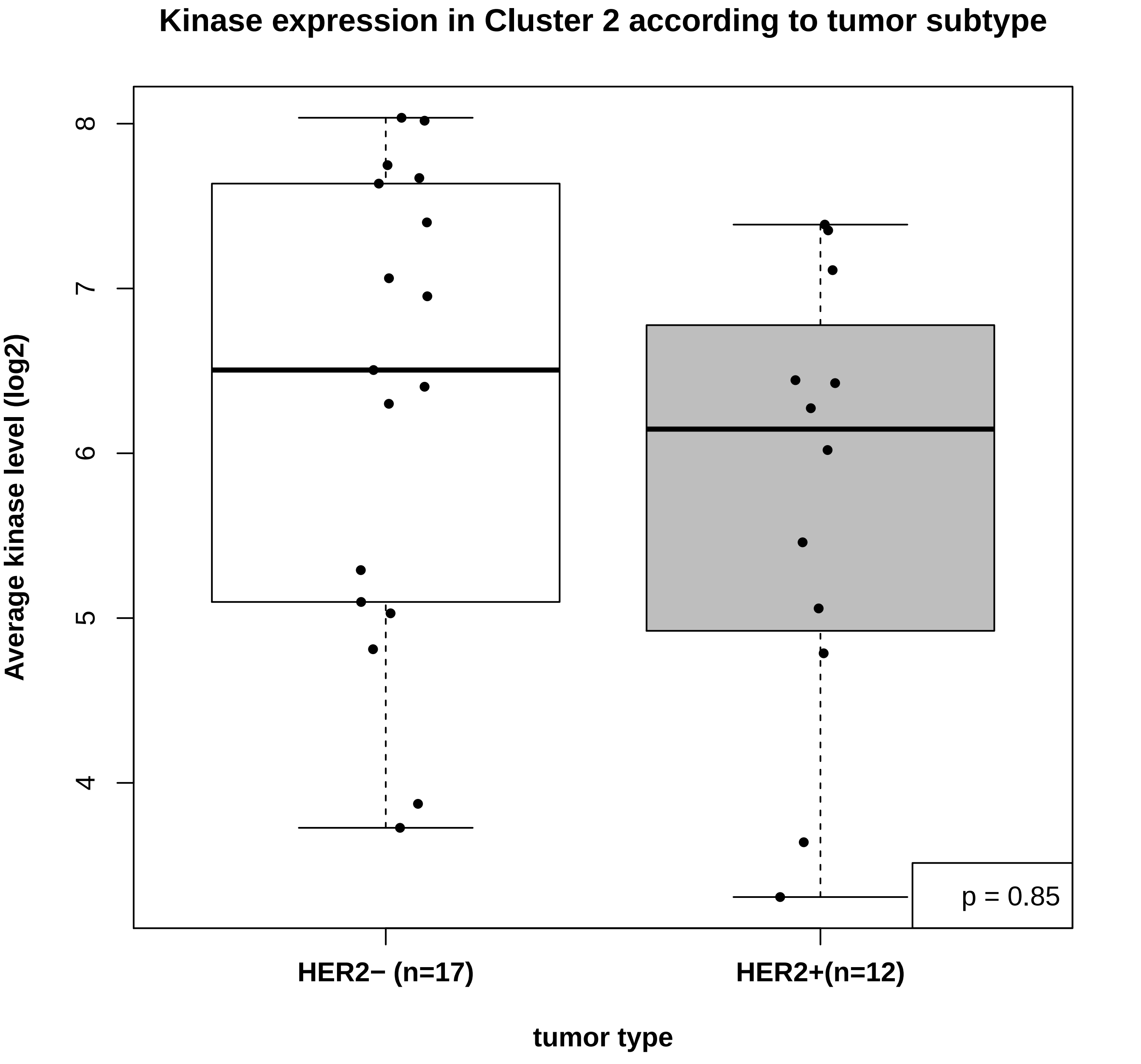
Kinase expression in Cluster 2 according to tumor subtype
Average kinase level (log2)
8
7
6
5
4
p = 0.85
HER2− (n=17)
HER2+(n=12)
tumor type

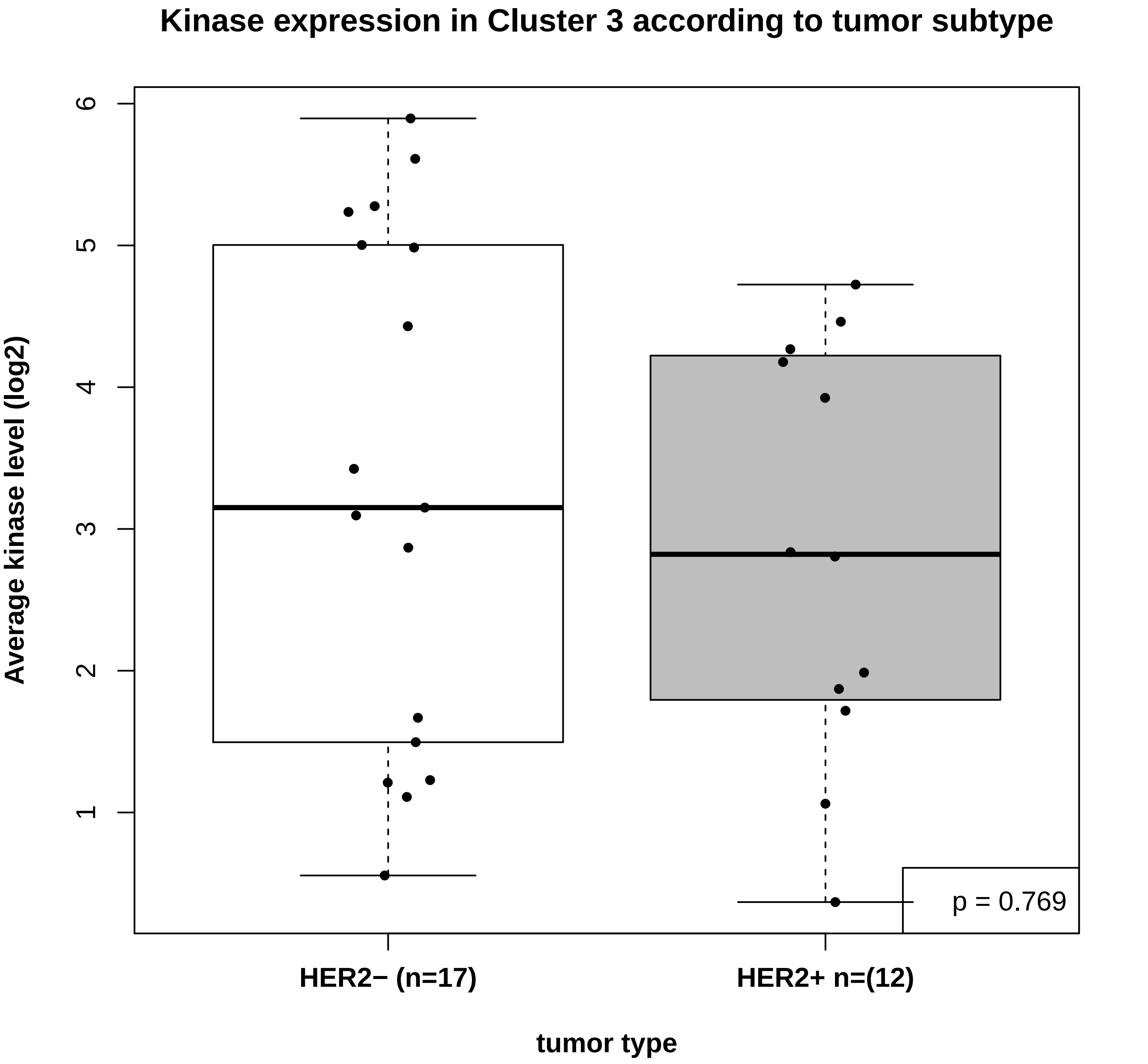
Kinase expression in Cluster 3 according to tumor subtype
Average kinase level (log2)
1
2
3
4
5
6
p = 0.769
HER2− (n=17)
HER2+ n=(12)
tumor type

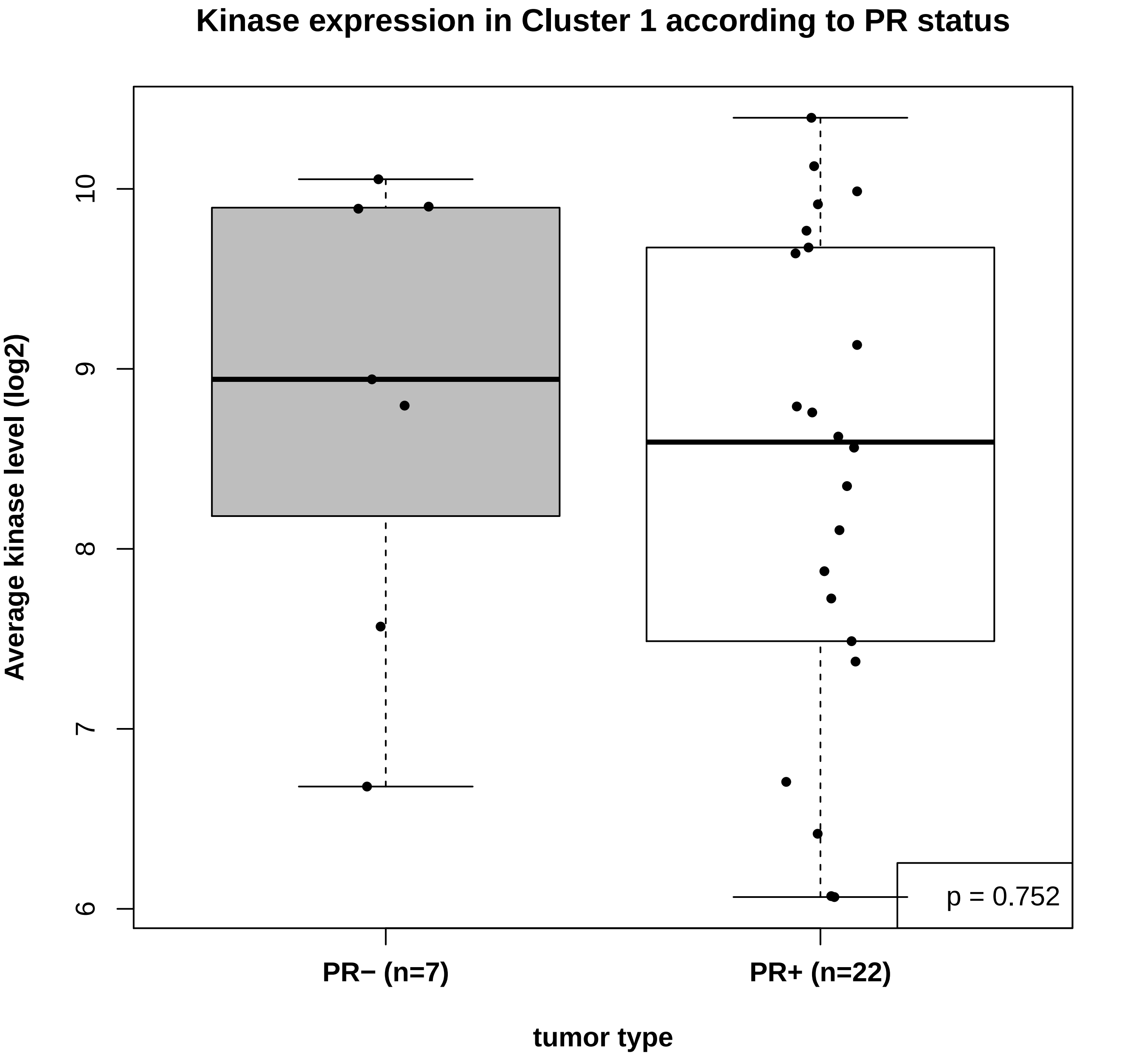

Kinase expression in Cluster 1 according to PR status
Average kinase level (log2)
10
9
8
7
6
PR− (n=7)
PR+ (n=22)
tumor type
p = 0.752

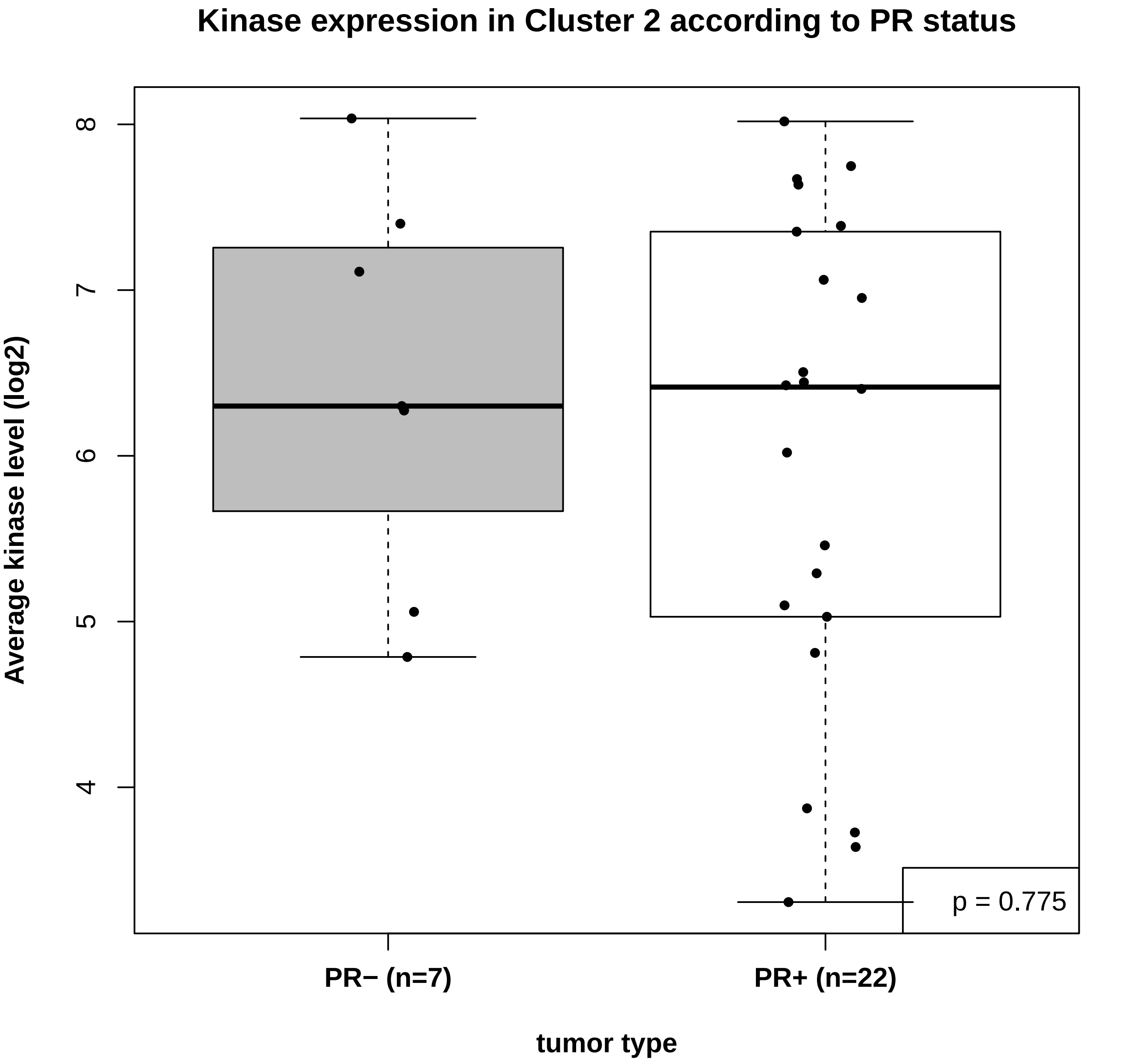

Kinase expression in Cluster 2 according to PR status
Average kinase level (log2)
8
7
6
5
4
p = 0.775
PR− (n=7)
PR+ (n=22)
tumor type

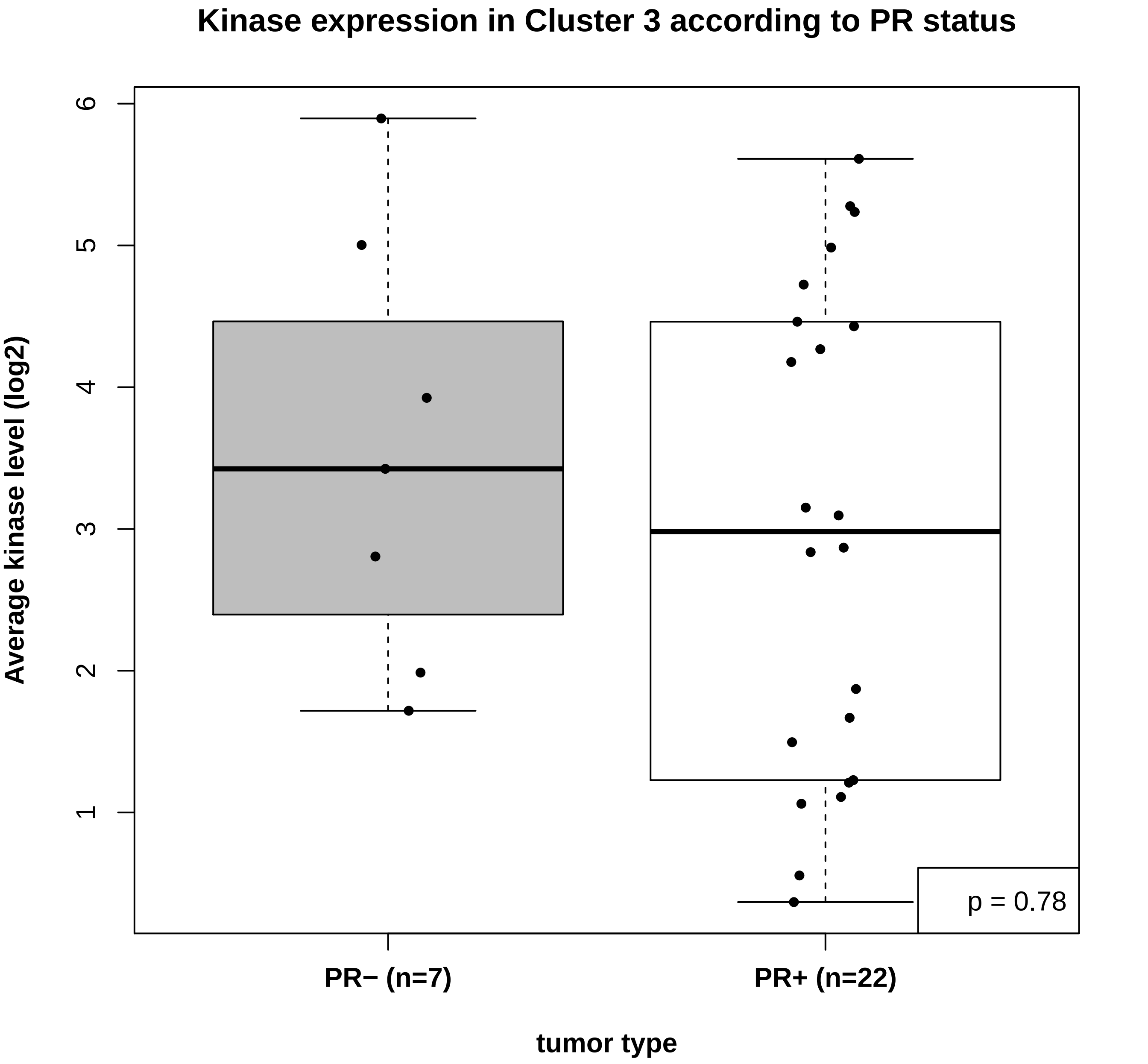
Kinase expression in Cluster 3 according to PR status
Average kinase level (log2)
1
2
3
4
5
6
p = 0.78
PR− (n=7)
PR+ (n=22)
tumor type
