# Supplementary material for: Loss of progesterone receptor is associated with distinct tyrosine kinase profiles in breast cancer
Source: Breast Cancer Res Treat. 2020 Jul 24;183(3):585–98. doi: 10.1007/s10549-020-05763-7 (PMC7497693; doi:10.1007/s10549-020-05763-7)
Supplement: Supplementary file 6 — Supplementary file6 (PDF 686 kb) [file 10549_2020_5763_MOESM6_ESM.pdf]

	Before adding estradiol			After adding es			Ratio (E10-Ctrl)		
	Mean	Mean	Mean	Mean	Mean	Mean	Ratio	Ratio	Ratio
**Peptide ID**	**BT474_E0**	**MCF7_E0**	**ZR75-1_E0**	**BT474_E10**	**MCF7_E10**	**ZR75-1_E10**	**BT474**	**MCF7**	**ZR75-1**
41_654_666	-0,51	-0,87	1,16	-0,49	-0,39	1,10	0,03	0,48	-0,06
ACHD_383_395	-0,04	-0,20	0,14	-0,08	0,08	0,09	-0,04	0,27	-0,05
AMPE_5_17	-0,07	-0,16	0,14	-0,16	0,14	0,10	-0,09	0,29	-0,05
ANXA1_14_26	-0,51	-0,67	1,11	-0,42	-0,47	0,96	0,09	0,21	-0,15
ANXA2_17_29	-0,42	-0,69	1,23	-0,45	-0,67	1,00	-0,02	0,02	-0,24
B3AT_39_51	-0,02	-0,11	0,22	-0,16	-0,25	0,33	-0,14	-0,14	0,10
C1R_199_211	0,11	-0,02	-0,39	0,29	-0,01	0,02	0,18	0,01	0,41
CALM_95_107	0,19	-0,10	-0,30	0,19	0,28	-0,26	0,00	0,38	0,04
CBL_693_705	-0,25	-0,59	0,54	-0,12	-0,09	0,51	0,13	0,50	-0,03
CD3Z_116_128	-0,56	-1,02	1,54	-0,69	-0,75	1,49	-0,13	0,27	-0,06
CD3Z_146_158	-0,41	-0,66	0,93	-0,53	-0,47	1,14	-0,12	0,19	0,21
CDK2_8_20	-0,77	-0,99	1,67	-0,86	-0,72	1,66	-0,09	0,28	-0,02
CDK7_157_169	-0,30	-0,22	0,48	-0,35	0,04	0,35	-0,05	0,25	-0,13
CRK_214_226	0,23	-0,25	0,12	-0,02	-0,21	0,13	-0,25	0,04	0,00
CTNB1_79_91	-0,38	-0,68	1,08	-0,43	-0,53	0,94	-0,05	0,15	-0,14
DCX_109_121	-0,30	-0,38	0,60	-0,39	-0,15	0,62	-0,09	0,23	0,02
DDR1_506_518	-0,40	-0,15	0,41	-0,50	0,17	0,46	-0,10	0,32	0,05
DYR1A_212_224	0,09	0,05	-0,51	0,01	0,17	0,19	-0,08	0,12	0,70
DYR1A_312_324	-0,30	-0,19	0,37	-0,32	0,10	0,34	-0,02	0,30	-0,03
EGFR_1103_1115	-0,52	-0,73	1,25	-0,55	-0,60	1,15	-0,03	0,13	-0,09
EGFR_1118_1130	0,20	0,11	-0,55	0,39	0,03	-0,18	0,20	-0,08	0,37
EGFR_1165_1177	-0,04	-0,30	0,40	-0,02	-0,42	0,38	0,02	-0,12	-0,02
EGFR_1190_1202	0,25	-0,04	-0,44	0,31	0,21	-0,29	0,06	0,26	0,15
EPHA1_774_786	-1,09	-1,07	2,03	-1,07	-0,79	2,00	0,02	0,28	-0,04
EPHA2_765_777	-0,98	-1,05	1,91	-0,99	-0,69	1,80	-0,01	0,36	-0,11
EPHA4_589_601	0,08	-0,08	0,06	0,04	0,06	-0,16	-0,05	0,14	-0,22
EPHA7_607_619	-0,80	-0,90	1,51	-0,71	-0,55	1,45	0,08	0,35	-0,06
EPHB1_771_783	-0,73	-0,86	1,52	-0,76	-0,54	1,36	-0,03	0,32	-0,16
EPHB1_921_933	-0,19	-0,16	0,23	-0,36	0,17	0,31	-0,18	0,33	0,08
EPHB4_583_595	0,00	0,03	-0,07	-0,01	0,08	-0,02	-0,01	0,05	0,05
EPOR_361_373	-0,63	-0,80	1,39	-0,75	-0,50	1,28	-0,13	0,30	-0,11
EPOR_419_431	-0,54	-0,87	1,45	-0,62	-0,81	1,38	-0,08	0,06	-0,07
ERBB2_1241_1253	-0,11	-0,66	0,67	-0,15	-0,39	0,65	-0,04	0,27	-0,01
ERBB2_870_882	-0,34	-0,61	0,75	-0,36	-0,41	0,96	-0,02	0,20	0,21
ERBB4_1181_1193	0,51	0,25	-0,27	0,43	-0,16	-0,75	-0,08	-0,41	-0,48
ERBB4_1277_1289	0,16	-0,28	0,31	0,25	-0,06	-0,37	0,09	0,22	-0,69
FAK1_569_581	-0,27	-0,33	0,40	-0,15	-0,04	0,39	0,12	0,28	-0,01
FAK2_572_584	-0,29	-0,45	0,61	-0,23	-0,20	0,56	0,05	0,24	-0,05
FER_707_719	-0,64	-0,87	1,47	-0,61	-0,69	1,34	0,03	0,18	-0,13
FES_706_718	-0,78	-0,99	1,71	-0,86	-0,71	1,63	-0,07	0,28	-0,08
FGFR1_761_773	0,05	-0,23	0,21	0,01	-0,08	0,04	-0,04	0,15	-0,17
FGFR2_762_774	-0,03	-0,45	0,42	0,06	-0,15	0,15	0,09	0,30	-0,27
FGFR3_753_765	-0,04	-0,56	0,56	0,02	-0,26	0,28	0,07	0,30	-0,28
INSR_1348_1360	0,16	0,08	-0,16	0,05	0,06	-0,20	-0,11	-0,02	-0,04
INSR_992_1004	0,21	-0,07	-0,21	0,32	0,14	-0,39	0,11	0,20	-0,18
JAK1_1015_1027	-0,40	-0,69	1,03	-0,50	-0,44	1,01	-0,10	0,24	-0,02
JAK2_563_577	-0,42	-0,59	0,94	-0,49	-0,32	0,87	-0,07	0,27	-0,08
K2C6B_53_65	-0,25	-0,34	0,51	-0,19	-0,07	0,34	0,06	0,27	-0,17
K2C8_425_437	-0,15	-0,30	0,58	-0,09	-0,36	0,33	0,06	-0,06	-0,25
KSYK_518_530	0,17	-0,17	-0,24	0,20	0,05	-0,01	0,03	0,23	0,22
LAT_194_206	-0,08	-0,41	0,27	-0,11	-0,01	0,35	-0,03	0,40	0,08
LAT_249_261	-0,37	-0,55	0,76	-0,48	-0,16	0,81	-0,11	0,38	0,05
LCK_387_399	-0,60	-0,85	1,44	-0,57	-0,68	1,27	0,03	0,17	-0,17
MBP_198_210	-0,32	-0,21	0,46	-0,37	-0,07	0,51	-0,05	0,14	0,05
MBP_259_271	-0,06	-0,15	0,02	-0,05	-0,07	0,32	0,01	0,08	0,30
MBP_263_275	-0,15	-0,05	0,25	-0,21	0,00	0,17	-0,06	0,05	-0,08
MET_1227_1239	-0,36	-0,61	0,91	-0,32	-0,44	0,82	0,04	0,18	-0,08
MK01_180_192	-0,09	-0,18	0,22	-0,03	-0,10	0,19	0,07	0,08	-0,03
MK07_211_223	-0,33	-0,04	0,44	-0,30	-0,04	0,27	0,03	0,00	-0,16
MK10_216_228	-0,31	-0,35	0,62	-0,34	-0,13	0,50	-0,03	0,22	-0,12
MK12_178_190	-0,05	-0,26	0,25	-0,02	-0,15	0,23	0,03	0,11	-0,01
MK14_173_185	0,14	-0,26	0,20	0,08	-0,22	0,06	-0,05	0,04	-0,14
NCF1_313_325	-0,34	-0,34	0,56	-0,37	0,01	0,46	-0,03	0,35	-0,10
NPT2A_501_513	-0,31	-0,21	0,44	-0,35	0,04	0,39	-0,04	0,25	-0,04
NTRK2_696_708	-0,40	-0,63	1,03	-0,48	-0,44	0,91	-0,08	0,19	-0,12
ODBA_340_352	0,14	-0,37	0,05	0,05	0,00	0,12	-0,09	0,37	0,07
P85A_600_612	-0,82	-1,17	1,94	-0,94	-0,87	1,86	-0,12	0,30	-0,08
PAXI_24_36	-0,63	-0,89	1,53	-0,84	-0,61	1,45	-0,21	0,29	-0,08
PDPK1_2_14	-0,43	-0,55	0,85	-0,49	-0,23	0,86	-0,06	0,32	0,01
PDPK1_369_381	-0,64	-0,88	1,48	-0,66	-0,68	1,38	-0,01	0,20	-0,11
PECA1_706_718	-0,67	-1,05	1,67	-0,84	-0,71	1,60	-0,16	0,34	-0,08
PGFRB_1002_1014	-0,14	-0,52	0,58	-0,23	-0,30	0,61	-0,09	0,23	0,03
PGFRB_1014_1028	-0,21	-0,53	0,61	-0,20	-0,29	0,62	0,00	0,24	0,01
PGFRB_709_721	-0,18	-0,41	0,71	-0,34	-0,23	0,45	-0,16	0,18	-0,25
PGFRB_768_780	-0,38	-0,59	1,00	-0,38	-0,45	0,81	0,00	0,14	-0,20
PGFRB_771_783	-0,36	-0,52	1,04	-0,45	-0,52	0,81	-0,09	0,00	-0,22
PP2AB_297_309	0,06	-0,23	0,14	0,00	-0,04	0,07	-0,06	0,19	-0,07
PRGR_786_798	-0,04	-0,47	0,61	-0,18	-0,26	0,34	-0,14	0,21	-0,27
PRRX2_202_214	-0,29	-0,48	0,86	-0,38	-0,26	0,56	-0,09	0,22	-0,30
PTN11_539_551	-0,09	-0,08	0,13	-0,06	0,00	0,10	0,03	0,08	-0,03
RAF1_332_344	-0,42	-0,80	1,21	-0,60	-0,54	1,14	-0,18	0,26	-0,07
RASA1_453_465	-0,52	-0,79	1,26	-0,62	-0,49	1,15	-0,10	0,30	-0,11
RB_804_816	0,19	-0,03	-0,63	0,34	0,16	-0,04	0,15	0,20	0,58
RBL2_99_111	-0,16	-0,27	0,43	-0,27	-0,07	0,34	-0,11	0,20	-0,08
RET_1022_1034	-0,83	-0,91	1,74	-0,85	-0,75	1,61	-0,02	0,16	-0,13
RON_1346_1358	-0,19	-0,54	0,70	-0,25	-0,32	0,59	-0,06	0,22	-0,11
RON_1353_1365	-0,10	-0,38	0,46	-0,14	-0,24	0,40	-0,05	0,14	-0,07
STAT4_714_726	-0,26	-0,51	0,82	-0,28	-0,44	0,67	-0,02	0,07	-0,15
TEC_512_524	-0,35	-0,63	0,89	-0,30	-0,49	0,88	0,06	0,13	-0,02
TNNT1_2_14	1,16	0,62	-1,96	0,81	0,48	-1,11	-0,36	-0,14	0,86
TYRO3_679_691	-0,22	-0,42	0,59	-0,28	-0,16	0,49	-0,05	0,26	-0,11
VGFR1_1040_1052	-0,13	-0,46	0,55	-0,16	-0,19	0,40	-0,03	0,27	-0,15
VGFR1_1049_1061	-0,13	-0,17	0,22	-0,14	-0,01	0,22	-0,01	0,16	-0,01
VGFR1_1206_1218	0,17	-0,08	-0,14	0,17	0,05	-0,17	0,00	0,13	-0,03
VGFR1_1235_1247	0,11	-0,01	-0,30	0,05	0,16	0,00	-0,06	0,17	0,31
VGFR1_1326_1338	-0,09	-0,42	0,54	-0,20	-0,16	0,33	-0,11	0,25	-0,21
VGFR2_1046_1058	-0,12	-0,33	0,41	-0,13	-0,29	0,46	-0,02	0,04	0,05
VGFR2_1052_1064	-0,12	-0,37	0,41	-0,13	-0,22	0,43	-0,01	0,15	0,02
VGFR2_1168_1180	-0,05	-0,15	0,14	-0,02	-0,08	0,16	0,02	0,07	0,02
VGFR2_944_956	-0,30	-0,21	0,35	-0,26	0,05	0,37	0,04	0,27	0,02
VGFR2_989_1001	-0,73	-1,10	1,75	-0,78	-0,80	1,66	-0,05	0,31	-0,09
VGFR3_1061_1073	0,00	-0,17	0,29	-0,19	-0,08	0,15	-0,19	0,10	-0,14
VINC_815_827	0,00	-0,12	0,05	0,00	-0,05	0,12	0,00	0,07	0,08
ZAP70_485_497	-0,33	-0,77	1,04	-0,31	-0,53	0,90	0,01	0,24	-0,14

34/104 (33%) peptides had increased phosphorylation
70/104 (67%%) peptides had lower phosphorylation

96/104 (92%) higher phosphorylation
8/104 (7%) lower phosphorylation

30/104 (29%) peptideshad increased phosphorylation
74/104 (71%) peptides had lower phosphorylation

After treating cells with estrogen for 10 min

	Increased phosphorylation	Decreased phosphorylation
**BT474**	33 %	67 %
**MCF7**	92 %	7 %
**ZR75-1**	29 %	71 %

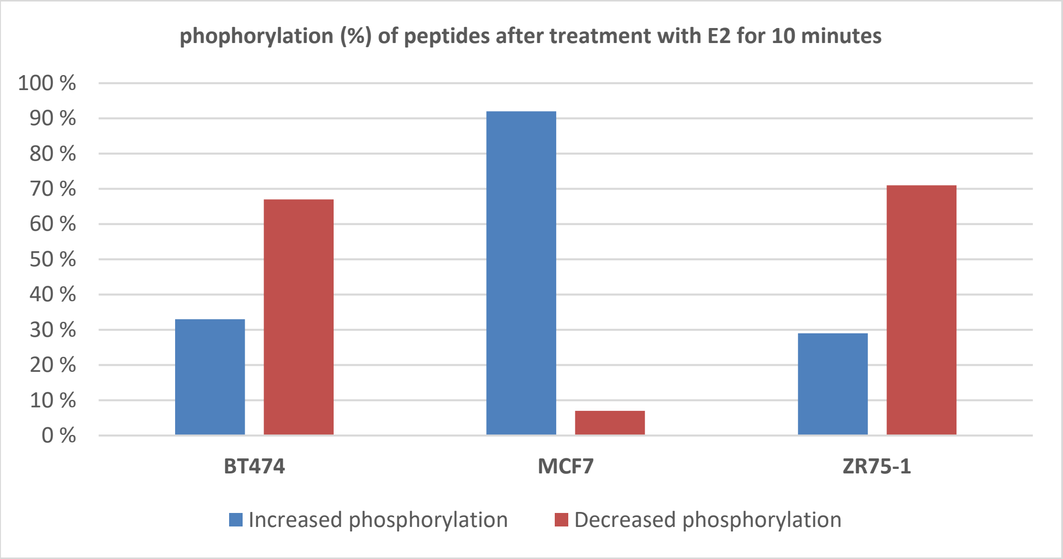

	p.value
**BT474 E0 vs E10**	4,742E-01
**MCF7 E0 vs E10**	2,712E-05
**ZR751 E0 vs E10**	6,671E-01

*** E0= no treatment with estradiol, E10 = treatment with estradiol for 10 minutes**
