# Supplementary material for: Loss of progesterone receptor is associated with distinct tyrosine kinase profiles in breast cancer
Source: Breast Cancer Res Treat. 2020 Jul 24;183(3):585–98. doi: 10.1007/s10549-020-05763-7 (PMC7497693; doi:10.1007/s10549-020-05763-7)
Supplement: Supplementary file 8 — Supplementary file8 (PDF 203 kb) [file 10549_2020_5763_MOESM8_ESM.pdf]

# overlaps shown:	50
# genesets in collections:	2199
# genes in comparison (n):	629
# genes in universe (N):	38055

**Gene Set Name**	**# Genes in Gene Set (K)**	**Description**	**# Genes in Overlap (k)**	**k/K**	**p-value**	**FDR q-value**
REACTOME_INTRAFLAGELLAR_TRANSPORT	54	Intraflagellar transport	12	0.2222	6.83E-11	1.5E-7
REACTOME_CILIUM_ASSEMBLY	201	Cilium Assembly	20	0.0995	1.91E-10	2.1E-7
REACTOME_ORGANELLE_BIOGENESIS_AND_MAINTENANCE	295	Organelle biogenesis and maintenance	22	0.0746	5.72E-9	4.19E-6
REACTOME_TRANSPORT_OF_SMALL_MOLECULES	728	Transport of small molecules	35	0.0481	2.53E-8	1.39E-5
REACTOME_CELL_CYCLE	642	Cell Cycle	31	0.0483	1.45E-7	6.37E-5
REACTOME_METABOLISM_OF_LIPIDS	738	Metabolism of lipids	33	0.0447	3.32E-7	1.21E-4
KEGG_PURINE_METABOLISM	159	Purine metabolism	14	0.0881	4.6E-7	1.44E-4
REACTOME_CELL_CYCLE_MITOTIC	536	Cell Cycle, Mitotic	26	0.0485	1.34E-6	3.69E-4
**REACTOME_INNATE_IMMUNE_SYSTEM**	1104	Innate Immune System	41	0.0371	1.63E-6	3.99E-4
**REACTOME_SIGNALING_BY_RECEPTOR_TYROSINE_KINASES**	468	Signaling by Receptor Tyrosine Kinases	23	0.0491	4.38E-6	9.63E-4
REACTOME_MITOTIC_PROMETAPHASE	198	Mitotic Prometaphase	14	0.0707	6.2E-6	1.15E-3
REACTOME_NEURONAL_SYSTEM	411	Neuronal System	21	0.0511	6.28E-6	1.15E-3
**REACTOME_MHC_CLASS_II_ANTIGEN_PRESENTATION**	124	MHC class II antigen presentation	11	0.0887	7.22E-6	1.22E-3
PID_ERA_GENOMIC_PATHWAY	64	Validated nuclear estrogen receptor alpha network	8	0.1250	1.04E-5	1.64E-3
REACTOME_NEUTROPHIL_DEGRANULATION	478	Neutrophil degranulation	22	0.0460	1.94E-5	2.84E-3
REACTOME_SLC_MEDIATED_TRANSMEMBRANE_TRANSPORT	250	SLC-mediated transmembrane transport	15	0.0600	2.09E-5	2.87E-3
REACTOME_GENERIC_TRANSCRIPTION_PATHWAY	1218	Generic Transcription Pathway	40	0.0328	3.72E-5	4.63E-3
REACTOME_ESTROGEN_DEPENDENT_GENE_EXPRESSION	148	Estrogen-dependent gene expression	11	0.0743	3.82E-5	4.63E-3
REACTOME_METABOLISM_OF_NUCLEOTIDES	99	Metabolism of nucleotides	9	0.0909	4.02E-5	4.63E-3
PID_SHP2_PATHWAY	57	SHP2 signaling	7	0.1228	4.21E-5	4.63E-3
NABA_ECM_REGULATORS	238	Genes encoding enzymes and their regulators involved in the remodeling of the extracellular matrix	14	0.0588	4.85E-5	4.86E-3
REACTOME_TRANSMISSION_ACROSS_CHEMICAL_SYNAPSES	269	Transmission across Chemical Synapses	15	0.0558	4.86E-5	4.86E-3
REACTOME_NON_INTEGRIN_MEMBRANE_ECM_INTERACTIONS	59	Non-integrin membrane-ECM interactions	7	0.1186	5.28E-5	5.05E-3
KEGG_GALACTOSE_METABOLISM	26	Galactose metabolism	5	0.1923	5.99E-5	5.49E-3
REACTOME_HEMOSTASIS	674	Hemostasis	26	0.0386	7.11E-5	6.25E-3
REACTOME_ESR_MEDIATED_SIGNALING	219	ESR-mediated signaling	13	0.0594	8.15E-5	6.33E-3
PID_HNF3A_PATHWAY	44	FOXA1 transcription factor network	6	0.1364	8.24E-5	6.33E-3
PID_INSULIN_PATHWAY	44	Insulin Pathway	6	0.1364	8.24E-5	6.33E-3
REACTOME_GENE_EXPRESSION_TRANSCRIPTION	1486	Gene expression (Transcription)	45	0.0303	8.35E-5	6.33E-3
PID_A6B1_A6B4_INTEGRIN_PATHWAY	46	a6b1 and a6b4 Integrin signaling	6	0.1304	1.06E-4	7.79E-3
REACTOME_RHO_GTPASES_ACTIVATE_FORMINS	139	RHO GTPases Activate Formins	10	0.0719	1.12E-4	7.95E-3
REACTOME_DISEASE	1075	Disease	35	0.0326	1.33E-4	8.75E-3
REACTOME_SIGNALING_BY_NUCLEAR_RECEPTORS	262	Signaling by Nuclear Receptors	14	0.0534	1.35E-4	8.75E-3
REACTOME_TRANSCRIPTIONAL_REGULATION_BY_TP53	363	Transcriptional Regulation by TP53	17	0.0468	1.35E-4	8.75E-3
REACTOME_DISEASES_OF_GLYCOSYLATION	143	Diseases of glycosylation	10	0.0699	1.42E-4	8.85E-3
KEGG_COMPLEMENT_AND_COAGULATION_CASCADES	69	Complement and coagulation cascades	7	0.1014	1.45E-4	8.85E-3
REACTOME_ACTIVATION_OF_C3_AND_C5	7	Activation of C3 and C5	3	0.4286	1.5E-4	8.9E-3
REACTOME_RECRUITMENT_OF_NUMA_TO_MITOTIC_CENTROSOMES	94	Recruitment of NuMA to mitotic centrosomes	8	0.0851	1.7E-4	9.86E-3
KEGG_PROPANOATE_METABOLISM	33	Propanoate metabolism	5	0.1515	1.96E-4	1.11E-2
REACTOME_DEVELOPMENTAL_BIOLOGY	1104	Developmental Biology	35	0.0317	2.2E-4	1.2E-2
KEGG_PYRIMIDINE_METABOLISM	98	Pyrimidine metabolism	8	0.0816	2.27E-4	1.2E-2
KEGG_CELL_CYCLE	124	Cell cycle	9	0.0726	2.29E-4	1.2E-2
REACTOME_RESOLUTION_OF_SISTER_CHROMATID_COHESION	125	Resolution of Sister Chromatid Cohesion	9	0.0720	2.43E-4	1.24E-2
**REACTOME_CYTOKINE_SIGNALING_IN_IMMUNE_SYSTEM**	856	Cytokine Signaling in Immune system	29	0.0339	2.58E-4	1.29E-2
REACTOME_TRANSPORT_OF_CONNEXONS_TO_THE_PLASMA_MEMBRANE	20	Transport of connexons to the plasma membrane	4	0.2000	2.9E-4	1.42E-2
REACTOME_M_PHASE	392	M Phase	17	0.0434	3.32E-4	1.58E-2
REACTOME_SIGNALING_BY_MET	79	Signaling by MET	7	0.0886	3.38E-4	1.58E-2
KEGG_ACUTE_MYELOID_LEUKEMIA	57	Acute myeloid leukemia	6	0.1053	3.53E-4	1.62E-2
PID_ERBB4_PATHWAY	38	ErbB4 signaling events	5	0.1316	3.88E-4	1.74E-2
**BIOCARTA_CTLA4_PATHWAY**	22	The Co-Stimulatory Signal During T-cell Activation	4	0.1818	4.27E-4	1.86E-2
