# Supplementary material for: Loss of progesterone receptor is associated with distinct tyrosine kinase profiles in breast cancer
Source: Breast Cancer Res Treat. 2020 Jul 24;183(3):585–98. doi: 10.1007/s10549-020-05763-7 (PMC7497693; doi:10.1007/s10549-020-05763-7)
Supplement: Supplementary file 10 — Supplementary file10 (PDF 173 kb) [file 10549_2020_5763_MOESM10_ESM.pdf]

a)

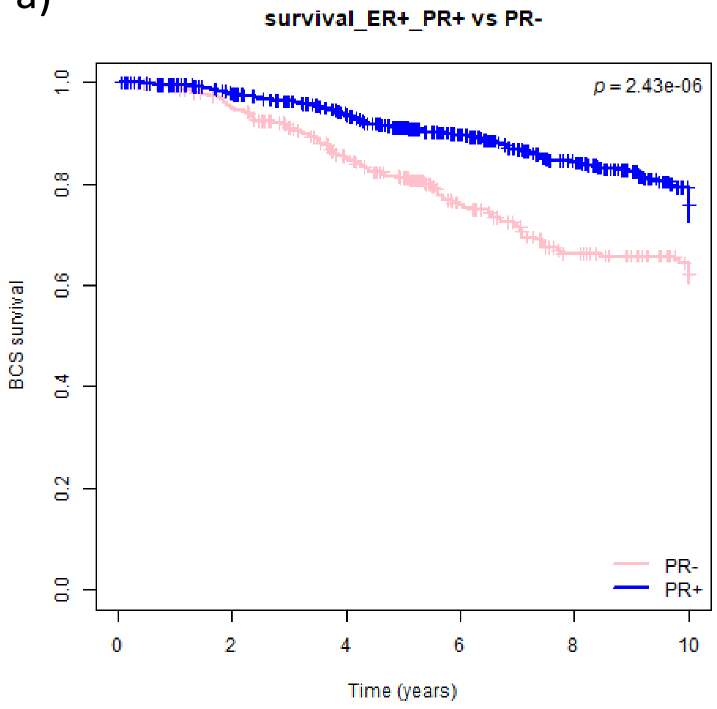

n= 1052 ER+ samples
ER+/PR+
ER+/PR-

b)

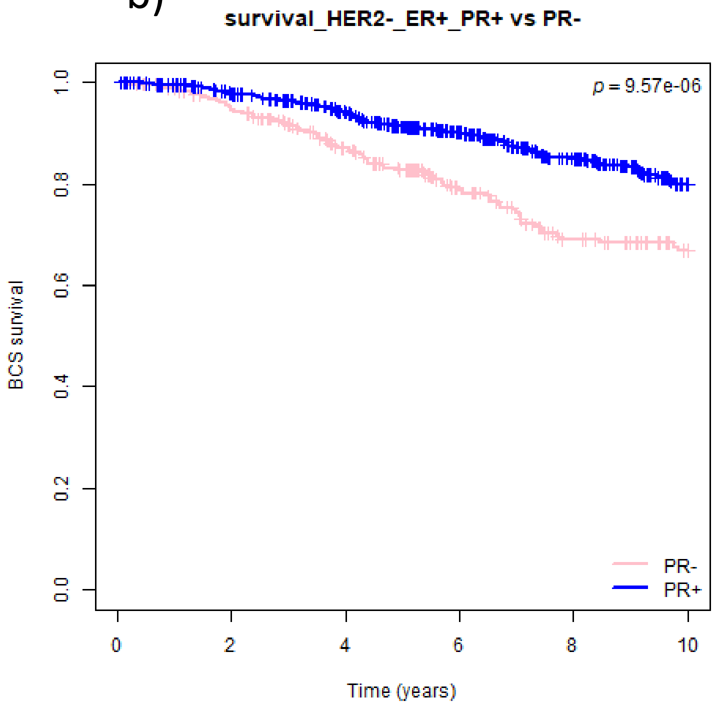

n= 977 ER+/HER2- samples
ER+/HER2-/PR+
ER+/HER2-/PR-

c)

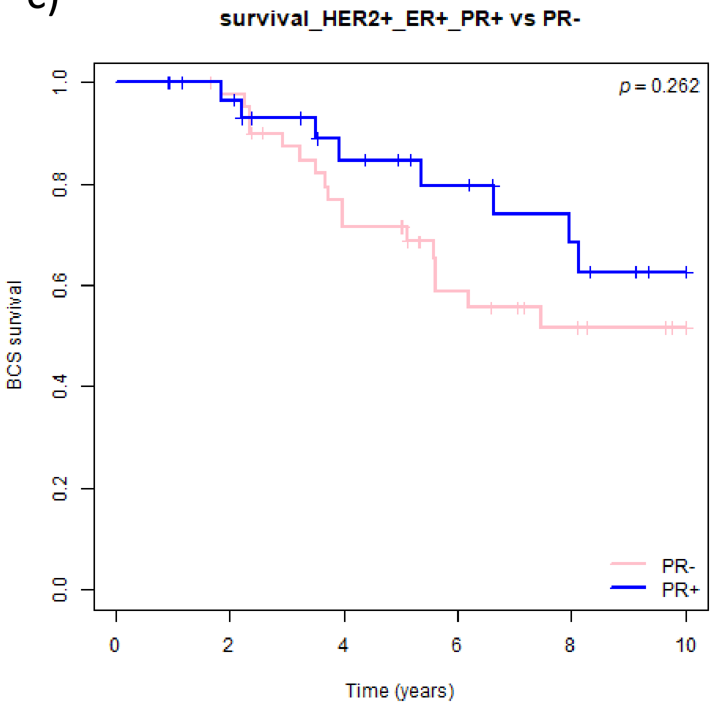

N = 75 ER+/HER2+ samples
ER+/HER2+/PR+
ER+/HER2+/PR-
